# A Genetic algorithm aided hyper parameter optimization based ensemble model for respiratory disease prediction with Explainable AI

**DOI:** 10.1371/journal.pone.0308015

**Published:** 2024-12-02

**Authors:** Balraj Preet Kaur, Harpreet Singh, Rahul Hans, Sanjeev Kumar Sharma, Chetna Sharma, Md. Mehedi Hassan

**Affiliations:** 1 Department of Computer Science and Engineering, DAV University, Jalandhar, Punjab, India; 2 Department of Computer Science and Engineering, Thapar Institute of Engineering and Technology, Patiala, India; 3 Department of Computer Science and Applications, DAV University, Jalandhar, Punjab, India; 4 Chitkara University Institute of Engineering and Technology, Chitkara University, Punjab, India; 5 Computer Science and Engineering Discipline, Khulna University, Khulna, Bangladesh; University of Electronic Science and Technology of China, CHINA

## Abstract

In the current era, a lot of research is being done in the domain of disease diagnosis using machine learning. In recent times, one of the deadliest respiratory diseases, COVID-19, which causes serious damage to the lungs has claimed a lot of lives globally. Machine learning-based systems can assist clinicians in the early diagnosis of the disease, which can reduce the deadly effects of the disease. For the successful deployment of these machine learning-based systems, hyperparameter-based optimization and feature selection are important issues. Motivated by the above, in this proposal, we design an improved model to predict the existence of respiratory disease among patients by incorporating hyperparameter optimization and feature selection. To optimize the parameters of the machine learning algorithms, hyperparameter optimization with a genetic algorithm is proposed and to reduce the size of the feature set, feature selection is performed using binary grey wolf optimization algorithm. Moreover, to enhance the efficacy of the predictions made by hyperparameter-optimized machine learning models, an ensemble model is proposed using a stacking classifier. Also, explainable AI was incorporated to define the feature importance by making use of Shapely adaptive explanations (SHAP) values. For the experimentation, the publicly accessible Mexico clinical dataset of COVID-19 was used. The results obtained show that the proposed model has superior prediction accuracy in comparison to its counterparts. Moreover, among all the hyperparameter-optimized algorithms, adaboost algorithm outperformed all the other hyperparameter-optimized algorithms. The various performance assessment metrics, including accuracy, precision, recall, AUC, and F1-score, were used to assess the results.

## 1. Background and rationale

**Fig 1:**
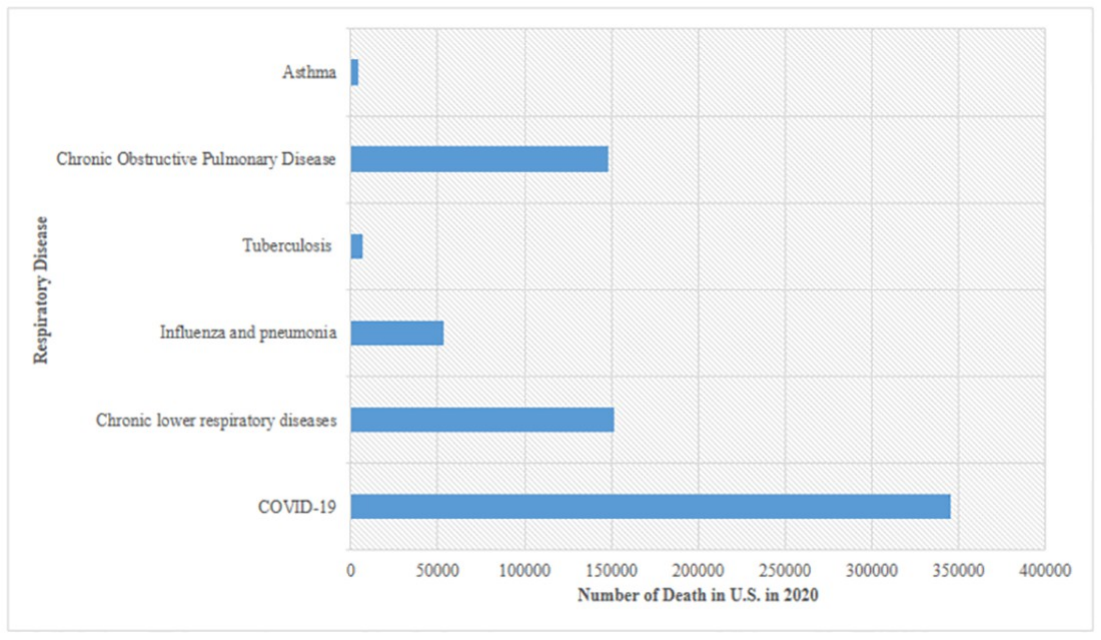
Mortality in U.S. in the year 2020 due to respiratory diseases.

Respiratory diseases are one of the main causes of mortality worldwide. Recently, one of the major respiratory diseases known as COVID-19, which has claimed a lot of lives globally, is one of the most disastrous pandemics seen by the human race in this century. The global lockdown and social distancing were new notions for the global population, living with these constraints was one of the most challenging tasks that changed the lifestyle of the population all around the world. The symptoms of this disease may vary from person to person depending upon the immunity of the body. The virus causing this deadly disease spreads to the respiratory tract of a person and causes grave damage to the lungs, which further leads to serious breathing problems which further reduces the oxygen level and requires further ventilator support for survival. COVID 19 alone has claimed over 6.5 million lives worldwide and diseases like chronic obstructive pulmonary disease (COPD) claim millions of lives every year worldwide [1]. Fig 1 shows the mortality in US alone in the year 2020 due to respiratory diseases.

While the World was battling with COVID-19, every possible avenue was explored to find the solution to this deadly disease [2]. To reduce the deadly effects of the disease, swift diagnosis of the disease is one of the most important factors to reduce the mortality rate due to this pandemic. Various diagnosis mechanisms, including real-time reverse transcriptase-polymerase chain reaction (RT-PCR), were taken into consideration for the diagnosis of the disease which appeared to be time time-consuming process [3]. Furthermore, different medical imaging systems, such as computed tomography (CT) and X-ray, can aid in swift diagnosis of the disease also new possibilities including artificial intelligence and big data can have been explored to control the spread of the pandemic [4].

In recent times, machine learning-based computer-aided diagnosis systems have come up as one of the most significant domains of research that assist radiologists in the accurate diagnosis of disease using medical images.

The ability of machine learning to adapt and learn from new data has enabled researchers to continuously refine strategies for managing and mitigating the impact of the disease, showcasing the potential of technology in addressing global health challenges [5]. Through the analysis of vast datasets, machine learning algorithms can be employed to predict the disease. Machine learning models have also been instrumental in developing diagnostic tools, such as predictive models for early detection of COVID-19 based on symptoms or imaging data [6].

In this context, this study aspires to utilize machine learning approaches and other clinical variables in the patient’s data for the development of a predictive model that can identify individuals with the existence of respiratory disease at an early stage and distinguish them from those who are healthy. Therefore, the main objective of this research is to assess and compare the outcome of the proposed model employing various hyperparameter tuning techniques with other state-of-the-art machine learning models.

### 1.1 Motivation

In recent times, machine learning has come up as one of the most promising domains of research proving its capability in the development of CAD systems for the diagnosis of various diseases [7]. However, for the impeccable deployment of these models, there is a huge room for improvement in various aspects viz. parameter tuning; for selecting the optimal parameters of the model that can lead to better results, and for feature selection; with an aim to reduce the dimensionality of dataset. This research considers the problem of respiratory disease classification and with an aim to improve the performance of existing machine learning algorithms, this research considers tuning the parameters of the algorithms and feature selection.

### 1.2. Problem identification

In this research, the authors aim to get into the bottom of two different problems required for the successful deployment of machine learning based systems which are parameter tuning and feature selection. Each machine learning algorithm has a different number and types of operations involving the use of different parameters [8]. For the successful deployment of these algorithms, the values of these parameters must be tuned to get the optimal set of values with an aim to achieve better classification accuracy. The problem of parameter tuning is regarded as an optimization problem that tries to optimize the various parameters to get the best set of parameters that aid in getting better accuracy [9]. The second problem considered by the researcher is feature selection, in which the authors try to reduce the dimensionality of the dataset by considering the most pertinent features and removing all the redundant features with an aim to increase the classification accuracy. Feature selection is also regarded as a multi-objective optimization problem that involves two different objectives viz. maximizing classification accuracy and minimizing the number of features [10].

Both the problems are complex optimization problems with different natures and require to be addressed differently to find the best solutions within a bearable time frame. Keeping in mind these goals an integrated system is required that simultaneously addresses all these issues and gives better classification accuracy.

### 1.3 Challenges and limitation of existing machine learning approaches in disease diagnosis

Machine learning (ML) has shown significant promise in disease diagnosis by automating and enhancing various aspects of the diagnostic process. However, there are several challenges and limitations that currently affect the efficacy and reliability of these approaches.

- Data quantity and quality.—High-quality, labeled medical data is scarce due to privacy constraints and the difficulty of obtaining sufficient cases for rare diseases, leading to data imbalances that bias machine learning models. Additionally, errors and inconsistencies in medical data from manual entry and diagnostic inaccuracies introduce noise, impairing model performance [11].
- Model related challenges.—Machine learning models in healthcare often overfit to training data, leading to poor generalization to new patients and variability across different settings, limiting their robustness. Many models, especially deep learning ones, are "black boxes," making their decisions difficult to interpret, which hampers clinical trust. Additionally, these models can perpetuate biases from training data, resulting in unfair treatment across diverse patient groups and raising ethical concerns about equity and fairness in medical outcomes [12].
- Implementation Challenges.—Integrating machine learning models into clinical workflows presents challenges, including the need for significant changes in how clinicians operate and manage data, alongside potential resistance from users due to trust issues and concerns about job security. ML models require extensive validation in clinical settings, a process that is costly and time-consuming, compounded by complex regulatory requirements [13].
- Other Challenges.—Training and deploying machine learning models, especially deep learning ones, demands significant computational resources, which can be a constraint for many healthcare facilities, particularly when real-time processing is required. Despite advancements, manual feature engineering remains essential for capturing domain-specific knowledge, a process that is both labor-intensive and dependent on expertise. Selecting relevant features from complex medical data is also crucial but challenging for model performance.

This study integrates advanced machine learning techniques with a framework based on SHapley Additive ExPlanations (SHAP) to address the limitations mentioned earlier, significantly enhancing the accuracy of COVID-19 diagnostic predictions. Genetic Algorithms (GAs) are employed for hyperparameter optimization due to their efficiency and effectiveness in locating optimal solutions [14]. By simulating the principles of natural selection, GAs thoroughly explore the hyperparameter search space, which helps in developing superior machine learning models with a high likelihood of reaching the global minimum and avoiding local minima [15]. For feature selection, the binary grey wolf algorithm is used, drawing inspiration from the behavior of grey wolves during round-up and hunting. The algorithm incorporates four types of grey wolves—alpha, beta, delta, and omega—to emulate the leadership hierarchy [16]. The optimization process includes the three main steps of hunting. searching for prey, encircling prey, and attacking prey, which are applied to enhance the model’s performance.

### 1.4 Research contributions

The contribution of the proposed research is fourfold; which has been summarized in the points below.

- Firstly, the algorithms’ hyperparameter tuning is proposed to enhance the efficacy of the machine learning classification algorithms.
- Secondly, an ensemble learning model is developed considering the performance of the various parameter-tuned classification algorithms.
- Thirdly, to select the most relevant feature nature-inspired metaheuristic algorithm is considered for reducing the dimensionality of the dataset to increase the classification accuracy in a bearable time.
- Lastly, to comprehensively analyze the observations’ prediction outcomes and interpret the justification behind the model’s classification decisions, SHAP analysis is performed.

### 1.5 Structuring of the paper

The rest of the article is structured as follows. Section 2 presents a concise overview of state-of-the-art research in the domain of disease detection using machine learning. The proposed model is presented in section 3. Section 4 describes the hyperparameter tuning with the Genetic algorithm. Section 5 briefly describes the dataset considered in this research. Section 6 presents experimental results and discussions. Feature importance using Explainable AI (SHAP Analysis) is discussed in section 7. Section 8 briefly presents the conclusions and future work.

## 2. Literature survey

This section summarizes the applications of machine learning in the domain of disease diagnosis, more specifically the diagnosis of COVID-19. Alali et al. [17] developed a highly efficient GPR-driven model to forecast the number of COVID-19 cases. The authors employed Bayesian optimization to fine-tune the hyperparameters of the Gaussian process regression in their model. Yank et al. [18] focused on enhancing the hyperparameters of well-known machine learning algorithms. Kumar et al. [19] presented an enhanced machine learning paradigm for the early detection of this illness. Modern Harris hawks optimization (HHO) algorithms based on random forest (HHORF), light gradient boosting (HHOLGB), extreme gradient boosting (HHOXGB), categorical boosting (HHOCAT) and support vector classifier (HHOSVC) were used to maximize the hyperparameters of the machine learning algorithms.

Mohsen et al. [20] used the generalized weighted ensemble with internally tuned hyperparameters (GEMITH) as a nested optimization-based technique that considers the tuning of hyperparameters and determining optimal weights for combining ensembles. Moreover, a heuristic approach was utilized to generate diverse and effective base learners, while Bayesian search was employed to expedite the optimization procedure.

Mohana et al. [21] used deep learning techniques on 350 images from X-ray datasets, the histogram equalization method was used for image preprocessing, and convolution neural network designs like ResNet-50 and VGG-16 were used for image categorization. The results indicated that, VGG-16 results in greater test and train precision. Further, to improve the results, hyper parameter optimization was used to fine-tune the VGG-16’s precision.

Soufiane et al. [22] presented the effectiveness of five different machine learning algorithms, namely Random Forest, Ada Boost, XGBoost, SVM and Decision Tree. For training and evaluation in the first experiment, each model used default parameters. In the second trial, the author’s employ the Grid Search function to identify the model’s ideal setup on a collection of anonymous individuals with or without COVID-19 illness. Aljouie et al. [23] employed four widely used machine learning methods, along with three data balancing approaches and feature selection techniques. Mohammad et al. [24] used a variety of machine learning techniques to predict the mortality rate among COVID-19 patients.

Many researchers have considered the use of feature selection techniques [25] for the diagnosis of the disease, which have been summarized in this section. Mehrdad et al. [26] introduced a new method for diagnosing COVID-19 that combines feature selection with random forest. The proposed method enhances the feature space, simplifies complexity, and provides clinicians with a decision tree-like analysis, facilitating easier explanation. Experimental results demonstrated that the developed prediction model surpassed existing methods and baseline algorithms in terms of performance. Fatih et al. [27] presented a novel approach for detecting COVID-19 automatically, employing a combination of fused dynamic exemplar pyramid feature extraction and hybrid feature selection techniques using deep learning. Extensive testing on various datasets demonstrated the method’s ability to achieve a high level of accuracy in detecting COVID-19. Chattopadhyay et al. [28] created various methods for COVID-19 detection, but only a few of them produced acceptable findings. The study makes two contributions, i.e., extracting deep features from the image dataset before introducing a totally new feature selection method called Clustering-based Golden Ratio Optimizer (CGRO).

Kenway et al. [29] suggested a framework which was divided into three stages that are linked together. Initially, features are extracted from CT images using the Convolutional Neural Network (CNN) known as AlexNet. Next, a feature selection method called Guided Whale Optimization (Guided WOA) is employed, which is based on Stochastic Fractal Search (SFS). Pramanik et al. [30] proposed a computer-aided diagnosis (CAD) system for detecting Pneumonia from chest X-rays, employing deep learning and a metaheuristic algorithm. The approach involved extracting deep features from a pre-trained ResNet50 model, which is fine-tuned on a specific Pneumonia dataset. The proposed method is evaluated using well-known UCI datasets, gene expression datasets based on microarray analysis, and a dataset for predicting COVID-19. Yagin et al. [31] discusses a study that utilizes machine learning techniques, specifically the XGBoost algorithm, to classify and assess COVID-19 patients based on genomic biomarkers. The model aims to provide a clear interpretation of individualized and overall risk estimation for COVID-19, aiding physicians in understanding the impact of key genomic features. The study highlights the importance of external validation, integration of clinical risk factors, and the need for multi-center trials to enhance the predictive accuracy of the model. Additionally, the use of Local Interpretable Model-Agnostic Explanations (LIME) and SHapley Additive exPlanations (SHAP) frameworks improves the accuracy of COVID-19 diagnostic prediction and aids in explaining predictions to clinicians. Hamal et al. [32] presents a study on using machine learning models to classify COVID-19-associated lung changes from X-ray images. The research evaluates various models and identifies VGG-19 with data augmentation as the top performer, achieving high precision, recall, and F1 scores for COVID-19, pneumonia, and healthy individuals. The study emphasizes the importance of image pre-processing, tuning, and augmentation in enhancing model performance. Héberger et al. [33] addresses common errors in statistical modeling and focuses on the significance of using performance parameters correctly. It highlights the importance of distinguishing between linear and nonlinear models in modeling processes. The study involves a multicriteria decision-making process to compare various modeling equations and optimization algorithms. It emphasizes the role of variance analysis in detecting outliers and underscores the necessity of data preprocessing. Table 1 presents the comparison of the prominent techniques in the literature.

**Table 1:** Comparison of key techniques in their literature.

Author	Dataset Type	Feature Selection	Hyperparameter Tuning	Technique Used	Future work
Dewi et al. [34]	csv	Boruta Feature Selection	Hyperband Optimization	Random Forest, XGBoost, Ensemble Methods	Combining different types of data (e.g., imaging, clinical, and genomic) can improve model robustness.
Soufiane et al. [22]	csv	No	Grid Search	Random Forest, Ada Boost, XGBoost, SVM and Decision Tree	Feature selection techniques and meta heuristic can be used for better performance.
Mehrdad et al. [26]	csv	Fisher score	No	XGBoost, SVM and MLP	Hyperparameter techniques and meta heuristic can be used for better performance.
Kumar et al. [19]	csv	No	Harris Hawks Optimization	Light gradient boosting, gradient boosting classifier, categorical boosting, random forest	More experiments can be conducted using feature selection methods to enhance model performance
Batista et al. [35]	csv	No	No	neural networks, random forests, gradient boosting trees, logistic regression and support vector machines	Feature selection techniques and Hyperparameter tuning can be used for better performance.
Chattopadhay et al. [28]	csv	Clustering-based Golden Ratio Optimizer	Wrapper-based FS algorithm	Support Vector Machines, K-Nearest Neighbor and Extreme Learning Machines.	Increased collaboration between generalizable models can lead to the development of more robust system.
Yasminah et al. [17]	csv	No	Bayesian Optimization	Support vector regression, Boosted trees, Bagged trees, Decision tree, Random Forest, and XGBoost	Feature selection techniques can be used for better performance.
Fatih et al. [27]	images	Local binary pattern	No	k-nearest neighbor	More models can be conducted to test performance.
Kukar et al. [36]	csv	No	No	random forest, neural network, the extreme gradient boosting machine and support vector machines	Feature selection techniques and Hyperparameter tuning can be used for better performance.
Meza et al. [31]	csv	No	No	Random forest, logistic regression, support vector machine, multilayer perceptron (neural network), stochastic gradient descent, XGBoost, and Adaboost	Feature selection techniques and Hyperparameter tuning can be used for better performance.

## 3. Proposed model

Machine learning has become one of the most significant domains of research these days and has its applications in various domains. For the successful deployments of machine learning models, certain unaddressed issues that can be considered for improvement as mentioned in the problem identification section.

**Fig 2:**
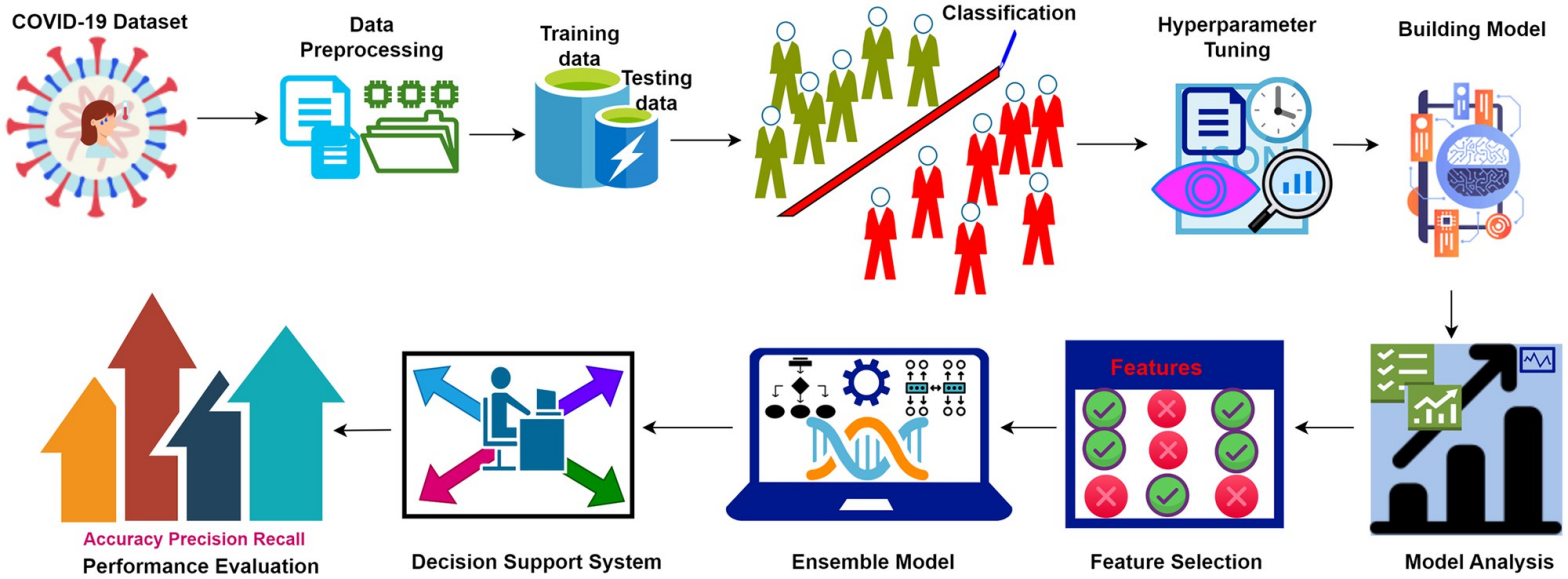
Proposed methodology.

In this light, authors in this research, aspire to use hyperparameters tuning and feature selection for machine learning algorithms to enhance the efficacy of the models. Fig 2 presents the primary steps in developing the proposed model.

### Step I- Preprocessing

This step aims to balance the dataset using upsampling techniques, as the COVID-negative cases constitute only 10.5% of the entire dataset, whereas positive samples make up 89.5% (refer to section 5). Following this, certain attributes are subsequently removed from metadata that is not related to the study goal, such as id, ID_Registro, Pecho_Acc, ABR_INT, Fecha_actulization, Ingreso, Fecha_DEF, Pias_origen and naciolandad, etc. Additionally, RESULTADO is taken into account to be a dataset class that contains COVID yes COVID no labels.

**Fig 3:**
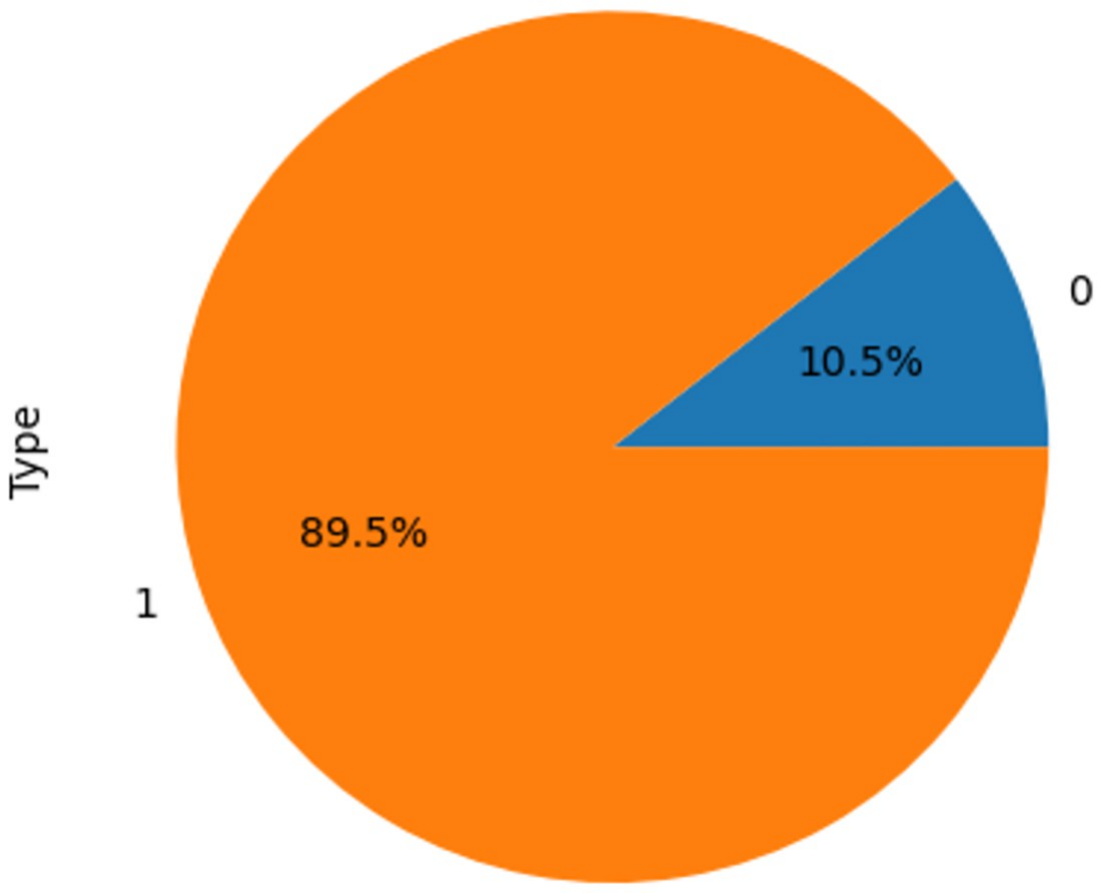
Before upsampling.

**Fig 4:**
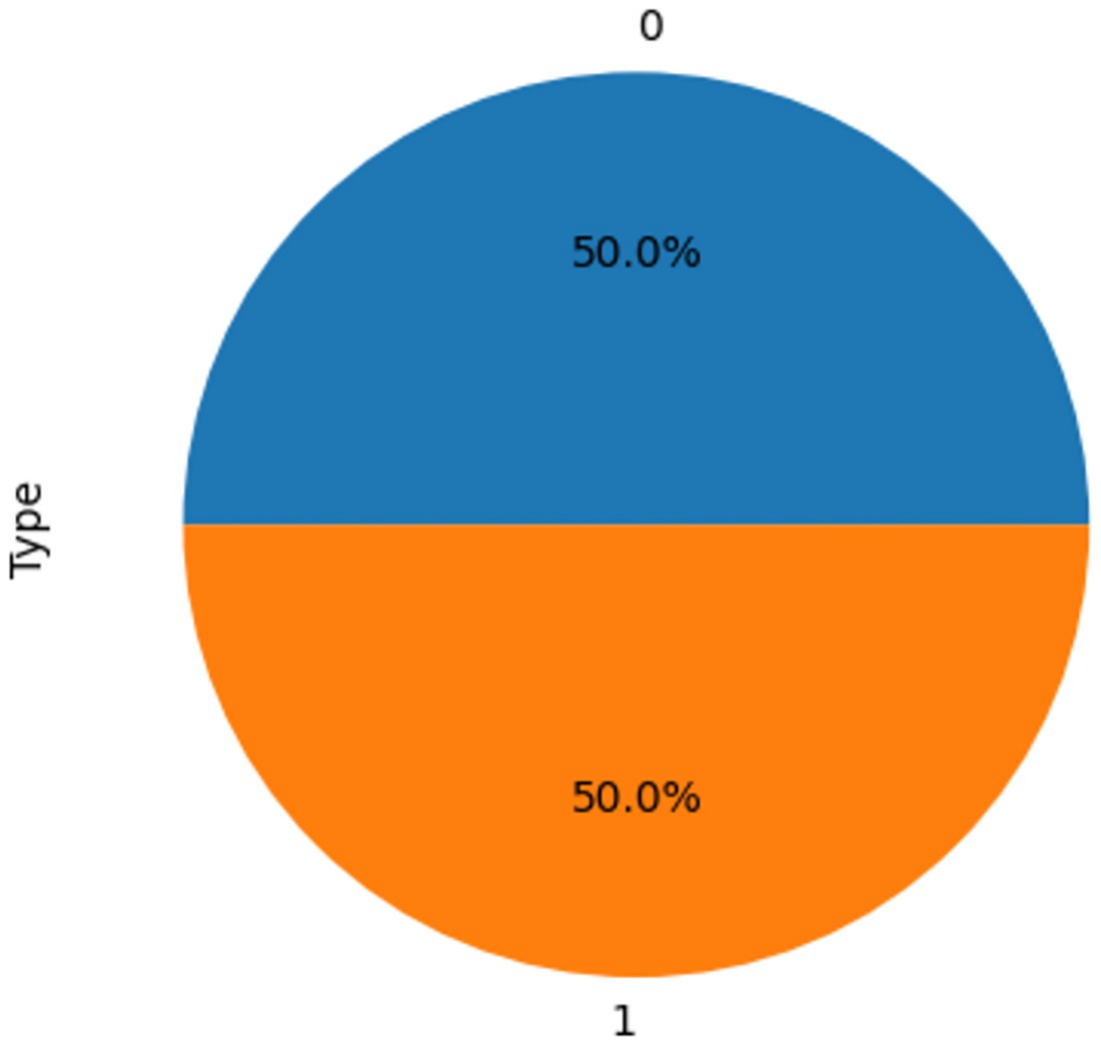
After upsampling.

As shown in Fig 3, before upsampling, the samples of the COVID-positive class accounted for 90 percent of the total, but after applying upsampling as shown in Fig 4, both the "yes" and "no" classes now possess an equal number of samples. Subsequent analyses and outcomes are based on this balanced dataset.

**Fig 5:**
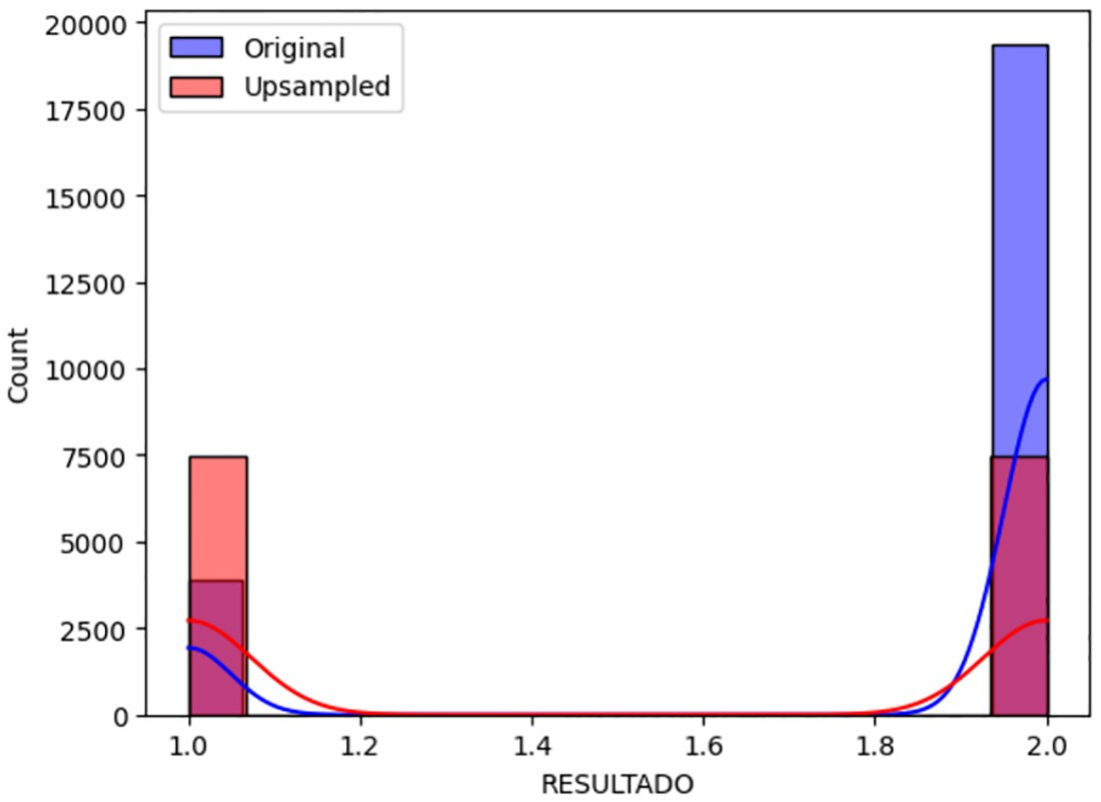
Comparison between original and upsampled dataset.

Original and after upsampling, a graph illustrating in Fig 5 the count of COVID-positive (Class 1) and COVID-negative (Class 0) cases reveals a balanced dataset. This balance is critical as it ensures equal representation of both classes, thereby enhancing the performance and reliability of the machine learning models. The graph underscores the effectiveness of upsampling in addressing class imbalance, a key factor in improving predictive accuracy and reducing model bias. The Mexico dataset was selected due to its extensive records on respiratory diseases and symptoms, which are highly correlated with COVID-19, providing a robust basis for analysis and comparison.

### Step II- Data splitting

For the training and assessment processes, the dataset ratios are 70% and 30%. Two tests were run. In the first, we used the models’ preset hyperparameters for training and testing. The confusion matrix was then created after we had computed the success measures. Secondly, classification results were taken with hyperparameter tuning.

### Step III- Classification algorithm

The presented system used seven classifiers. Adaboost, Random forest, Extra tree, Decision Tree, Gradient Boosting Classifier, KNN and Light Gradient Boosting Machine.

### Step IV- Hyperparameter tuning

Numerous machine learning applications in the actual world heavily rely on hyperparameter optimization. The hyperparameters of these algorithms can be optimized to boost the efficiency of these algorithms. Genetic algorithms, random search, Bayesian Optimization, and grid search are used as optimization methods. The various hyperparameters used by various classifiers are.

LightGBM. num leaves, bagging fraction, feature fraction, learning rate, max depth, subsample, colsample tree, max bin, min child samples.

Adaboost. (subsample, colsample tree, gamma, max depth, min child weight, learning rate, alpha).

Random Forest. (n estimator, criterion, max depth, min sample split).

The efficiency of the categorization can be improved by carefully choosing (tuning) the values of the hyperparameters. When an optimization algorithm is present, the tuning process can be completed, and the full process is referred to as an optimization issue.

### Step V- Building and model analysis

The performance was evaluated by considering the confusion matrix with several metrics, including precision, accuracy, area under the curve, error rate, balanced accuracy score, cross-validation score, Kappa index, and F1-score. The 2X2 CM has been used in the suggested model hyperparameter optimization-based ML method to assess the model using the mentioned metrics. The results that were properly categorized are represented by the categorization along the main diagonal. (Higher numbers of the metrics, excluding the error stated above, indicate a more effective model.

### Step VI- Feature selection

**Fig 6:**
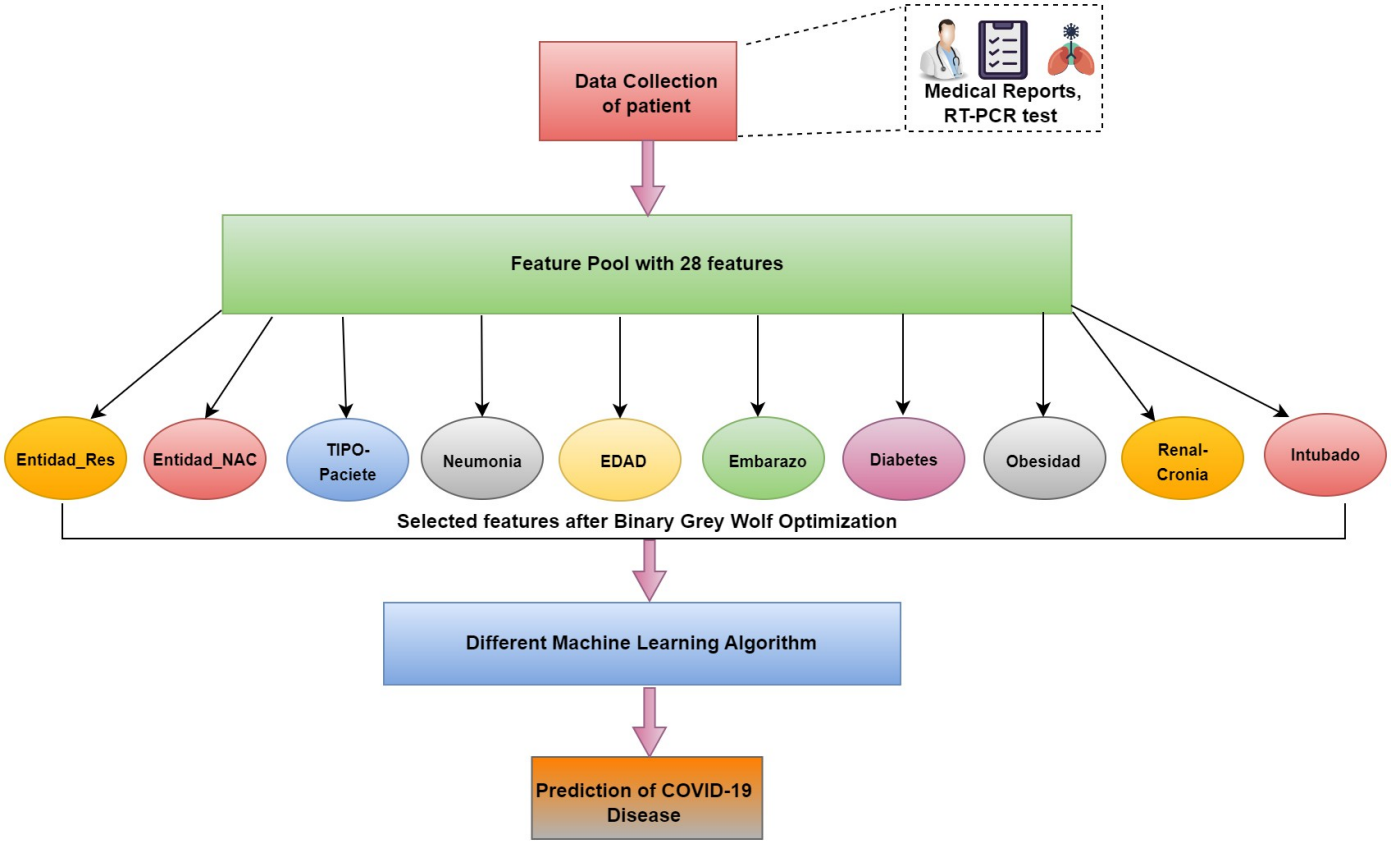
Feature selection process.

The proposed research uses one of the latest nature-inspired metaheuristic algorithms, "grey-wolf optimizer" that imitates the natural command structure and foraging strategy of grey wolves [37] for feature selection. The algorithm searches the space of features to find the best features from the original set of features with an aim to maximize the accuracy of prediction and minimize the number of features selected. Fig 6 presents the feature selection process considered in this research. Using feature selection, one can determine the crucial features and eliminate the unnecessary (redundant) ones from the dataset [38]. For various machine learning applications, the feature selection goals include reducing data dimensionality, enhancing prediction performance, and providing good data understanding [39].

### Step VII- Ensemble model

In the proposed model, stacking method [40] is used as ensemble learning. The strategy of this method is employed to enhance the predictive efficacy of machine learning models. This approach entails amalgamating several foundational models to construct a more robust meta-model that capitalizes on the distinct capabilities of each foundational model.

**Fig 7:**
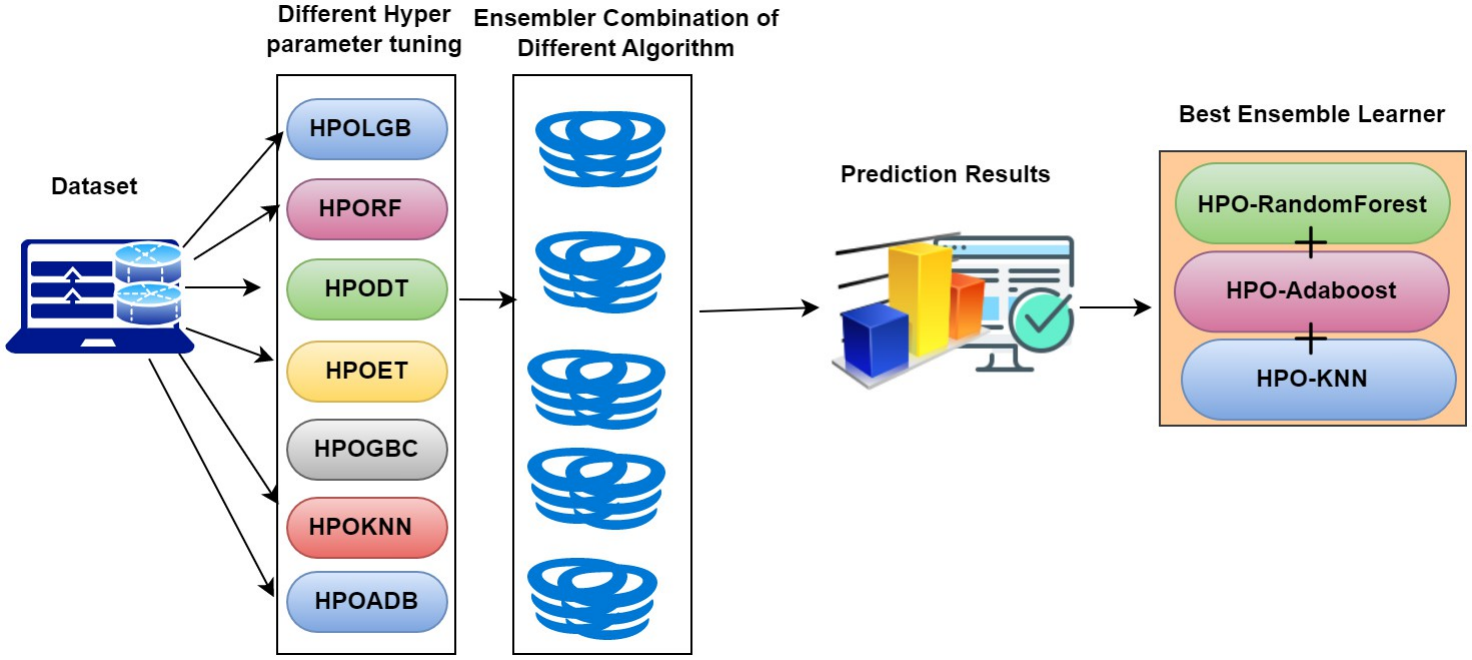
Ensemble model architecture.

The fundamental concept behind stacking revolves around incorporating one or more meta-level models, which accept predictions from multiple foundational models as inputs and subsequently generate the ultimate prediction. The greatest precision is achieved when Adaboost, KNN, and Random forest are combined as shown in Fig 7. The mathematical formula [41, 42] is demonstrated as Eq 1

$$y=mode\left\{cl1,cl2,cl3\right\}$$ (1)

y is the stacking classifier for getting the result by adding three machine learning with the best result.

### Step VIII- Performance evaluation

A performance assessment model enables precision and efficiency evaluations. There are numerous methods for rating classifiers. In this study, we utilized the Holdout technique, which involves partitioning the dataset into two separate subsets. a test set and a train set, with each comprising 30% and 70% of the dataset, respectively. The training process involved using the train set to train the data, and afterward, we assessed its predictive capabilities by evaluating it on the hidden test set [43]. To mitigate overfitting in the proposed model, a feature selection process was employed to eliminate noise and remove features that were either redundant or of minimal importance for prediction accuracy. Additionally, an ensemble modeling approach was adopted to further reduce overfitting. Ensemble methods enhance model performance by combining multiple weak learners, which collectively produce more accurate and robust results. By leveraging multiple models to analyze the data, ensemble techniques ensure that the final predictions are more reliable and precise. Additionally, we employed the Cross-validation technique to prevent the over-fitting issue. Then, we determined some assessment metrics, including the F1 score, ROC, memory, accuracy, and precision [44].

## 4. Hyperparameter tuning using Genetic algorithm

**Fig 8:**
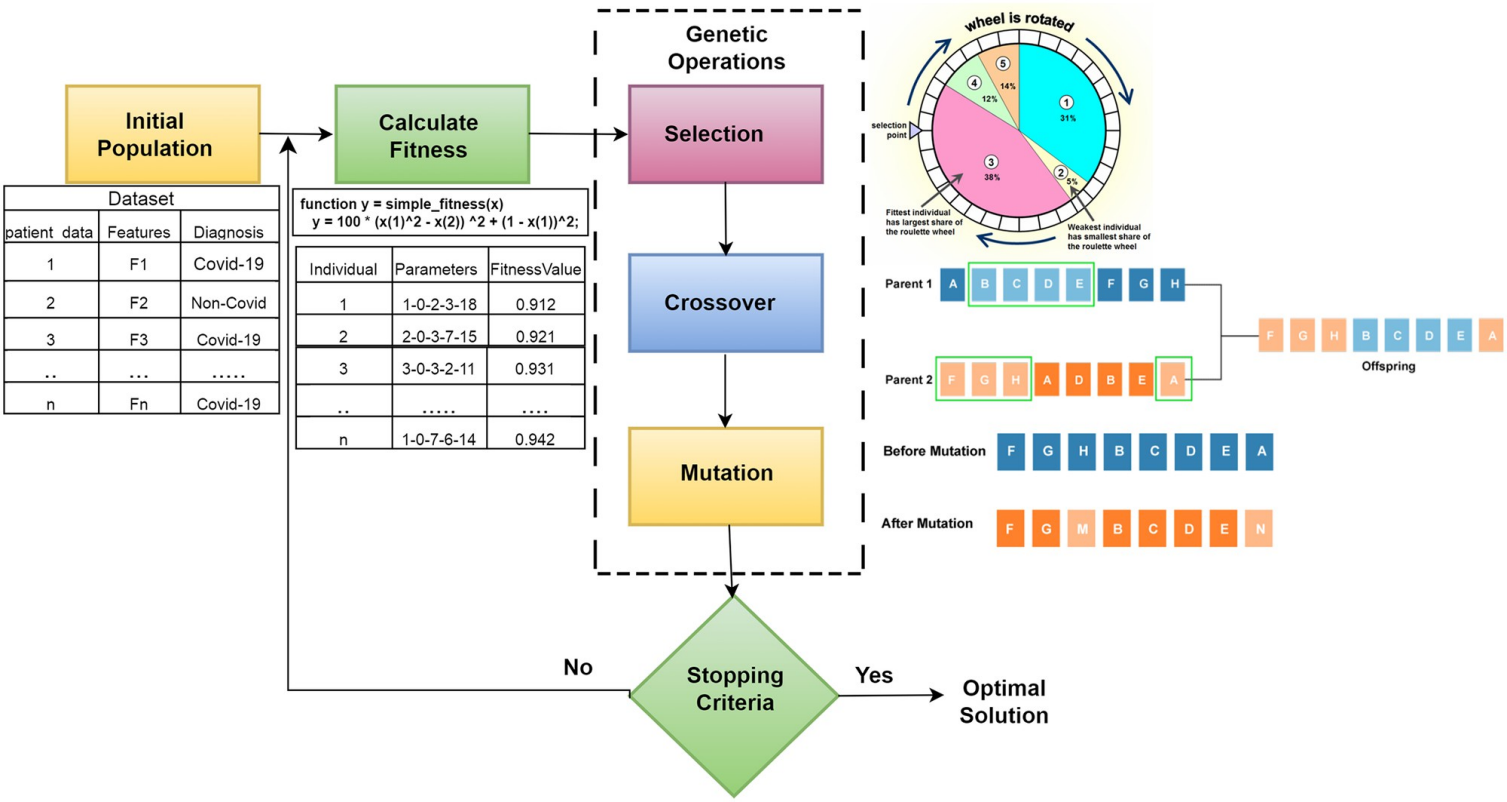
The framework of Genetic algorithm for hyper-parameter optimization.

Genetic algorithms can optimize machine learning algorithm’s hyperparameters by systematically exploring potential hyperparameter combinations. Fig 8 shows the structure of genetic hyperparameter tuning on a machine learning algorithm [45].

Start by identifying the hyperparameters to fine-tune the machine learning model, such as learning rates or layer sizes. For each hyperparameter, specify the range or values it can take.

### Population initialization

Begin with an initial set of hyperparameter combinations, forming a population of candidates. A population comprises individuals or solutions, typically referred to as chromosomes. Each chromosome is composed of a series of genes, where each gene, or multiple genes, depending on the encoding method, represents a single decision variable that will be applied to the objective functions. Various parameter combinations lead to diverse fitness values in vectors. Random mutations in the parameters are introduced within the population, and vectors with higher fitness levels outlive their counterparts [46].

#### Evaluation phase

Train and assess a machine learning model for each candidate hyperparameter set. Use a performance metric (e.g., accuracy, error) to quantify how well each model performs.

#### Selection

Choose a subset of candidates (called parents) for the next generation based on their model performance. Candidates who achieve better results are more likely to be selected. Common selection methods include random sampling or ranking candidates.

#### Crossover (Recombination)

Pair up the selected parents and generate new candidate hyperparameter sets (offspring) by merging their hyperparameter values. Crossover can involve blending or swapping hyperparameter values between parent candidates to create offspring.

#### Mutation

Introduce small, random changes to hyperparameters in some offspring candidates. This introduces diversity into the population. Mutation helps explore the hyperparameter space more extensively.

#### Population update

Replace some existing candidates with the newly generated offspring candidates. The selection for replacement is often based on the fitness (performance) of the candidates. This step ensures the population size remains consistent.

#### Termination conditions

Specify when to stop the optimization process. This can be based on a maximum number of iterations, a performance threshold, or a time limit.

#### Final result

The hyperparameter set that results in the best model performance during the optimization process is considered the optimal configuration [47].

The algorithm for generating hyperparameters using the genetic algorithm is as shown in Table 2.

**Table 2:** Algorithm for generating hyperparameter.

Algorithm. Genetic Algorithm for Hyperparameter Tuning
Result. The fittest hyperparameter in the population populations [list of n models with different hyperparameters generation 0; **while** generation<max generation **do** train_and_evaluate(population); new_gen ← retains the m fittest individuals; new_gen ← append random individuals to promote diversity; mutate(mew_gen); new_gen ← append offsprings through crossover until k; population ← new_gen; generation ← generation+1 **end**

Here’s a breakdown of how this process works.

1. **Generate Initial Population**. Begin by creating an initial set of machine learning (ML) models with randomly selected hyperparameters.
2. **Evaluate Loss Function**. Determine the loss function for each model, such as log-loss, to measure their performance.
3. **Select Top Models**. Identify and select a subset of models with the lowest error rates.
4. **Create Offspring**. Develop a new population of ML models by generating offspring from the top-performing models of the previous generation, making slight adjustments to their hyperparameters. Combine these offspring with models from the previous generation and new models in a specific ratio, for instance, 50/50.
5. **Iterate the Process**. Calculate the loss function for the new population, rank the models, and repeat the process for multiple generations.

Genetic algorithms, while powerful, require careful specification of the loss function, population size, and the ratio of offspring with modified parameters [48].

## 5. Dataset description

The dataset [49] contains 41 columns which includes clinical data as well as RT-PCR test. The 41 columns have certain attributes that aren’t required for the findings, thus they’re omitted from the dataset. Some non-relevant fields have been eliminated, including the patient’s ID, city name, and patient registration date, as well as nine additional columns. Table 3 shows that the 29 most relevant columns. Out of all the samples in the dataset, subjects having initial respiratory problems were considered for the study which make about 14964 patients’ samples [49]. The column’s values are the description of attributes. The value 1 means yes and the value 0 means no. Table 3 shows attributes’ descriptions as there are 29 main attributes consisting of personal attributes and clinical features [49].

**Table 3:** Dataset description.

S. No.	Attribute Name	Description
1.	Entidad_um	Region where hospital performed admission
2.	Entidad_Res	Residence of the patient at which region
3.	Delay	Lag in the process of lab report
4.	Entidad_Registro	The actual region from where case assigned
5.	Origen	surveillance of patient (1 = yes, 2 = no)
6.	Sector	identify the institute of national health system
7.	Sexo	gender of patient 1 = female, 2 = male and 99 for undisclosed
8.	Entidad_nac	patient birth state or region
9.	Tipo_paciente	type of care patient received (1 = outpatient, 2 = impatient)
10.	Neumonia	Identifies whether the patient was diagnosed with pneumonia
11.	Edad	Age of the patient
12.	Nacionalidad	check whether patient is Mexican(1) or foreign(2)
13.	Embrazo	Identifies patient is pregnant or not
14.	Habla_lengua_Indig	Patient speaks an indigenous language
15.	Diabetes	Identifies whether the patient was diagnosed with diabetes
16.	EPOC	Classify whether the patient detect with pulmonary disease
17.	Asma	Classify whether the patient diagnosed with asthma or not
18.	Immusupr	Identifies if the patient is immune suppressed
19.	Hipertension	Classify whether the patient diagnosed with hypertension
20.	Otra_Com	Identifies if the patient presents another disease
21.	Cardiovascular	Classify whether the patient diagnosed with cardiovascular disease or not
22.	Obesidad	Classify whether the patient diagnosed with obesity or not
23.	Renal_Cronica	Identifies if chronic renal insufficiency
24.	Tabaquismo	Identifies if tobacco addiction
25.	Otro_Caso	Classify whether the patient diagnosed with any other case diagnosed with SARS COV-2
26.	Migrante	Identifies if the patient is migrant
27.	UCI	Identifies if the patient was admitted to ICU
28.	Intubado	patient need intubation or not(1 = yes, 2 = no,97 = not applicable)
29.	Resultado	The RT-PCR test (1 = positive, 2 = negative)

## 6. Experimental results and discussions

In this section, experimental results obtained after implementation Binary Grey Wolf Optimization for feature selection on dataset. It significantly impacts machine learning model performance by enhancing predictive accuracy, reducing computational complexity, improving interpretability, and ensuring robust generalization. Its effective exploration and exploitation strategies enable the selection of optimal feature subsets, contributing to the development of more efficient, reliable, and scalable machine learning models [50]. Further experimental results obtained after implementation and execution of the proposed model are compared with other state of the art machine learning algorithms, which are decision tree, adaboost, random forest, gradient boosting, light gradient boosting, extra tree, logistic regression, ridge classifier, linear discriminant analysis, naïve bayes, K-nearest neighbor and support vector machine, based on different evaluation metrics like accuracy, precision, recall, f1-score, Kappa_stat, MCC, and time required [51].

To validate the results 3-fold cross validation technique is considered. Table 4 summarizes the results obtained by executing different parameters as mentioned above. The results indicate the outperformance of the decision tree classifier classification algorithm in terms of different evaluation parameters.

**Table 4:** Results of machine learning algorithm.

Model	Accuracy	AUC	Recall	Prec.	F1	Kappa	MCC	TT(Sec)
**Decision Tree Classifier**	**0.9593**	**0.9722**	**0.9926**	**0.9484**	**0.9700**	**0.9385**	**0.9396**	**0.0810**
Extra Trees Classifier	0.9564	0.9906	0.9935	0.9425	0.9673	0.9328	0.9342	1.1020
Random Forest Classifier	0.9544	0.9872	0.9962	0.9367	0.9655	0.9288	0.9307	1.2150
Gradient Boosting Classifier	0.9489	0.9821	0.9948	0.9283	0.9604	0.9179	0.9203	0.7160
Light Gradient Boosting Machine	0.9484	0.9819	0.9977	0.9250	0.9600	0.9167	0.9196	0.4200
Ada Boost Classifier	0.9475	0.9776	0.9877	0.9200	0.9583	0.9129	0.9165	0.4590
Logistic Regression	0.9469	0.9739	0.9861	0.9198	0.9582	0.9127	0.9163	1.0460
Naive Bayes	0.9454	0.9709	0.9776	0.9198	0.9582	0.9127	0.9163	0.0780
Ridge Classifier	0.9434	0.0000	0.9678	0.9198	0.9582	0.9127	0.9163	0.0650
Extreme Gradient Boosting	0.9384	0.9788	0.9647	0.9198	0.9582	0.9127	0.9163	1.0720
Linear Discriminant Analysis	0.9364	0.9734	0.9577	0.9198	0.9582	0.9127	0.9163	0.1010
SVM—Linear Kernel	0.9176	0.0000	0.9319	0.9093	0.9149	0.8352	0.8437	0.1390
K Neighbors Classifier	0.8300	0.9100	0.8862	0.7967	0.8390	0.6599	0.6644	0.1750
Quadratic Discriminant Analysis	0.5000	0.0000	0.8570	0.5000	0.6667	0.0000	0.0000	0.0800

**Fig 9:**
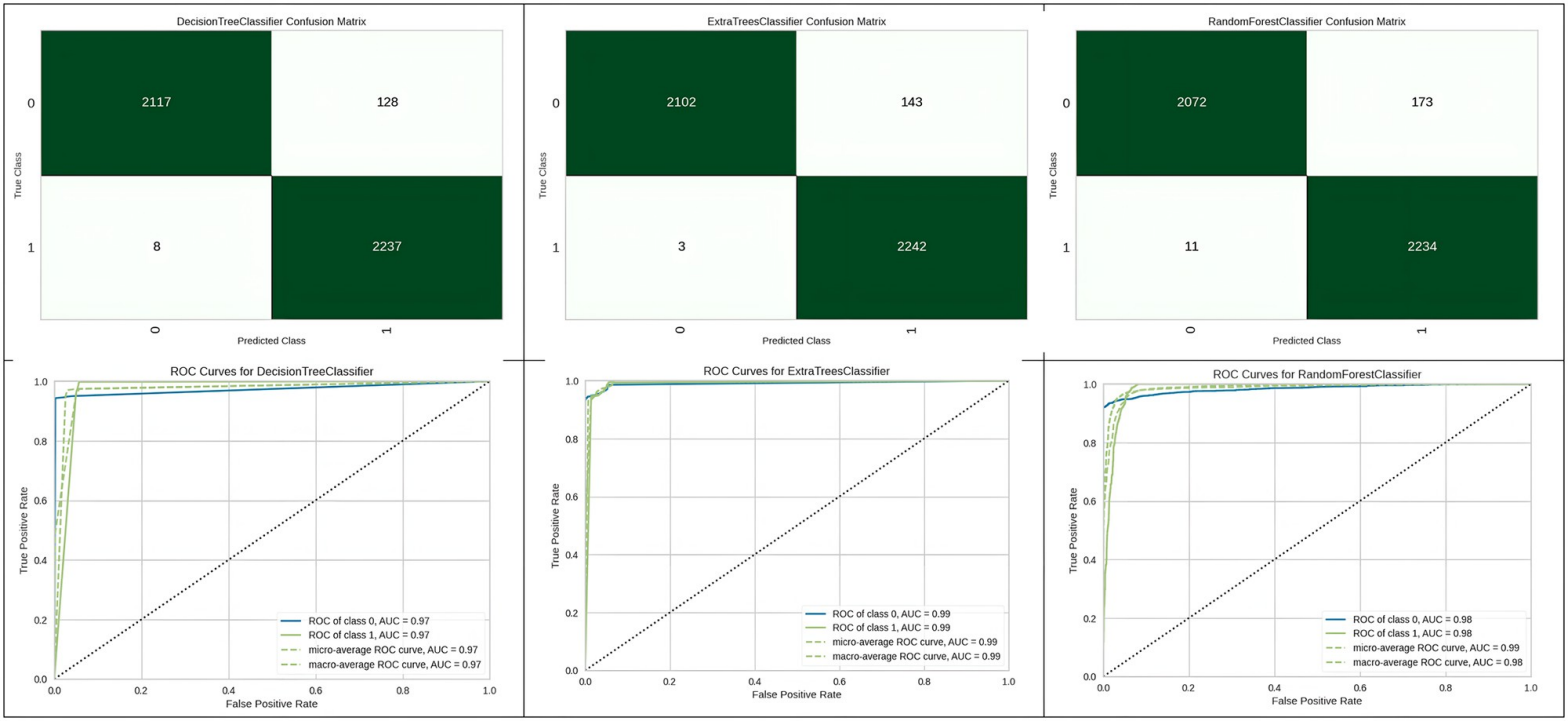
Best three classifier confusion matrix and ROC curve.

Fig 9 represents the confusion matrix obtained and the corresponding ROC curve for the top three best algorithms which are the Decision tree, Extra tree and Random Forest. The decision tree model achieved a sensitivity of 96%, a specificity of 92%, and a positive likelihood ratio of 18.19. In comparison, the Extra Trees model demonstrated a sensitivity of 95.5%, while the Random Forest model yielded a sensitivity of 94%, both of which are slightly lower than the sensitivity achieved by the decision tree.

Pondering further, seven different algorithms were considered for hyperparameter optimization using grid search, random search, and Bayesian Optimization and Genetic algorithm techniques. Table 5 represents the results obtained after performing the hyperparameter optimization. The results indicate that when hyperparameter optimization of random forest is performed using a genetic algorithm, the results indicate the outperformance of the algorithm as compared to other algorithms considered for hyperparameter optimization.

**Table 5:** Random forest with hyperparameter optimization.

S.no	Hyperparameter optimization Algo	Accuracy	Optimized parameter	Computation Time
1	Default Hyperparameters	0.952053729	max_depth = 2, random_state = 0	10.5s
2	Grid Search	0.9490457097	{’criterion’. ’gini’, ’max_depth’. 15, ’n_estimators’. 20}	6.06s
3	Random Search	0.9511253	{’max_depth’. 19,, ’min_samples_leaf’. 8, ’min_samples_split’. 10,criterion’. ’gini’, ’max_features’. 16, ’n_estimators’. 56’}	7.89s
**4**	**Genetic Algorithm**	**0.9585935**	**min_samples_split = 4,max_depth = 92, min_sample s_leaf = 6, max_features = 10, n_estimators = 92**	**15.8s**
5	Bayesian Optimization	0.948092	{’min_samples_leaf’. 4.0, ’max_depth’. 39.0, ’min_samples_split’. 3.0, ’criterion’. 1, ’n_estimators’. 97.0,’max_features’. 5.0}	30.5s

**Fig 10:**
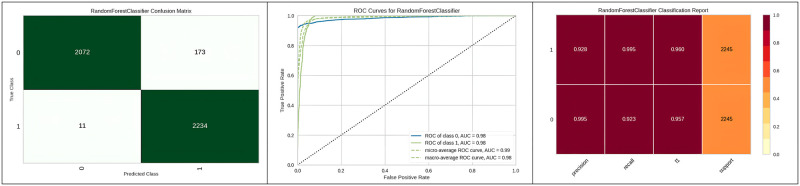
Best optimizer results for random forest.

Fig 10 represents the confusion matrix obtained, corresponding ROC curve and classification report of the random forest with genetic algorithm hyperparameter optimization.

Table 6 represents the results obtained after performing the hyperparameter optimization on the gradient boosting classifier. The results indicate that when hyperparameter optimization of gradient boosting classifier is performed using a genetic algorithm, the results indicate the outperformance of the algorithm as compared to other algorithms considered for hyperparameter optimization.

**Table 6:** Gradient boosting classifier results.

S.no	Hyperparameter optimization Algo	Accuracy	Optimized parameter	Computation Time
1	Default Hyperparameters	0.948578654	{’max_depth’.3,’random_state’. none, ’learning_rate’. 0.1, ’subsample’. 1.0, ’n_estimators’.100,}	110.5s
2	Grid Search	0.94905854	{’subsample’. 0.7,’learning_rate’. 0.1, ’n_estimators’.250,’random_state’. 1, ’max_depth’.3}	168.06s
3	Random Search	0.948323611	{’subsample’. 0.5, ’random_state’. 1,, ’max_depth’. 2, ’n_estimators’. 1000, ’learning_rate’. 0.1}	87.89s
**4**	**Genetic Algorithm**	**0.9521277**	**{’subsample’. 0.5, ’random_state’. 1, ’max_depth’. 2, ’n_estimators’. 745, ’learning_rate’. 0.01}**	**185.8s**
5	Bayesian Optimization	0.9501281	{’subsample’. 0.75, ’random_state’. 1, ’learning_rate’. 0.01,’n_estimators’. 1795, ’max_depth’. 1}	30.5s

**Fig 11:**
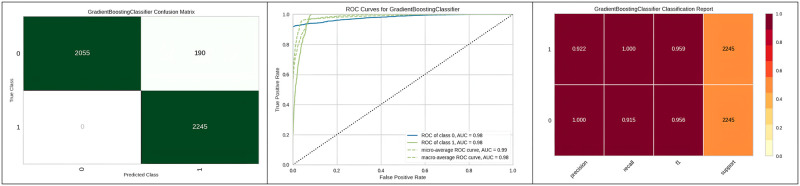
Best optimizer result for gradient boosting classifier.

Fig 11 represents the classification report, confusion matrix obtained and corresponding ROC curve of the gradient boosting classifier with genetic algorithm hyper parameter optimization. Furthermore, the outperformance of the Adaboost classifier with the genetic algorithm is represented in Table 7. The computation time of random search is often higher than other methods due to its time complexity.

**Table 7:** Adaboost classifier results.

S.no	Hyperparameter optimization Algo	Accuracy	Optimized parameter	Computation Time
1	Default Hyperparameters	0.94732345	learning_rate = 1.0, algorithm = ’SAMME.R’, n_estimators = 50	160.5s
2	Grid Search	0.94724405	{’algorithm’. ’SAMME.R’, ’n_estimators’. 4, ’learning_rate’. 1.01}	178.06s
3	Random Search	0.9462440	{’learning_rate’. 0.99, ’algorithm’. ’SAMME.R’, ’n_estimators’. 7}	197.89s
**4**	**Genetic Algorithm**	**0.95696142**	**{’learning_rate’. 1.02, ’algorithm’. ’SAMME.R’, ’n_estimators’. 8}**	**135.8s**
5	Bayesian Optimization	0.94912440	{’n_estimator’. 20, ’learning rate’. 1.02, algorithm. ’SAMME’}	260.5s

**Fig 12:**
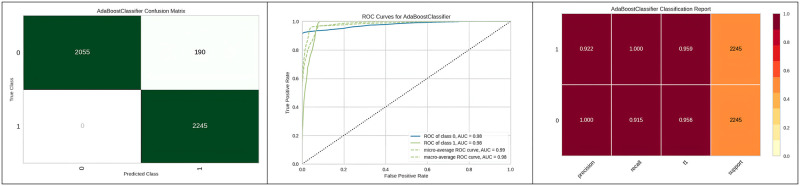
Best optimizer result for Adaboost classifier.

Fig 12 represents the true positive and false positive values in the confusion matrix and ROC curve of the Adaboost classifier with micro average and AUC. Table 8 represents the optimized parameters of Extra Trees using various optimization algorithms. Additionally, Table 8 illustrates the improved results of Extra Trees with the genetic algorithm optimization compared to other hyperparameter optimization algorithms.

**Table 8:** Results of Extra tree.

S.no	Hyperparameter optimization Algo	Accuracy	Optimized parameter	Computation Time
1	Default Hyperparameters	0.95631	{n_estimators = 100, *, max_depth = None, min_samples_split = 2, criterion = ’gini’}	160.5s
2	Grid Search	0.94428	{’max_features’. 3, ’n_estimators’. 100, ’min_samples_leaf’. 5}	178.06s
3	Random Search	0.948318	{’n_estimators’.200,’min_samples_leaf’. 5, ’max_features’. 4}	197.89s
**4**	**Genetic Algorithm**	**0.957013**	**{’n_estimators’.500, ’max_features’. 4, ’min_samples_leaf’. 5}**	**135.8s**
5	Bayesian Optimization	0.942421812	min_samples_leaf = 10, n_estimators = 300, max_features = 3	260.5s

**Fig 13:**
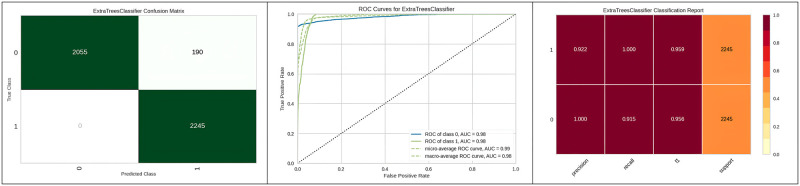
Best optimizer result for Extra tree.

Fig 13 represents the confusion matrix, ROC curve, and classification report of Extra Trees with genetic algorithm hyperparameter optimization, showcasing its higher performance compared to other optimization algorithms. Moving ahead, Table 9 represents the results obtained after performing the hyperparameter optimization on the Light gradient boosting machine. The results indicate that when hyperparameter optimization of the Light gradient boosting machine is performed using a grid search algorithm, the results indicate the outperformance of the algorithm as compared to other algorithms considered for hyperparameter optimization.

**Table 9:** Results of Lightbgm.

S.no	Hyperparameter optimization Algo	Accuracy	Optimized parameter	Computation Time
1	Default Hyperparameters	0.94845	(num_leaves = 31, max_depth = 1, learning_rate = 0.1, n_ colsample_bytree = 1.0, reg_lambda = 0.0, estimators = 100)	160.5s
**2**	**Grid Search**	**0.956261**	**(boosting_type = ’dart’, colsample_bytree = 0.6, learning_rate = 1, max_depth = 5, n_estimators = 20, num_leaves = 5, reg_lambda = 1)**	**158.06s**
3	Random Search	0.949122	{’reg_lambda’. 0.01, ’num_leaves’. 25, ’n_estimators’. 35, ’boosting_type’. ’gbdt’,’max_depth’. 15, ’colsample_bytree’. 1, ’learning_rate’.0.1}	197.89s
4	Genetic Algorithm	0.948192	(n_estimators = 35, boosting_type = dart,colsample_bytree = 1, max_depth = 5, num_leaves = 50, reg_lambda = 0.1, learning_rate = 0.1)	195.8s
5	Bayesian Optimization	0.947296	(learning_rate = 1, max_depth = 15, n_estimators = 35, num_leaves = 5, reg_lambda = 1, colsample_bytree = 0.6)	260.5s

**Fig 14:**
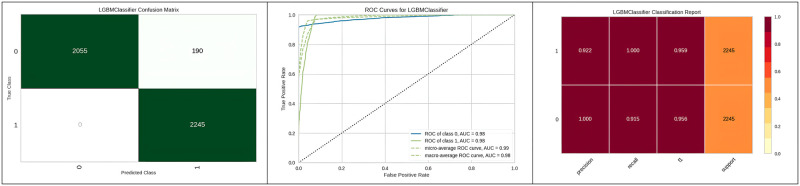
Best optimizer results of Lightbgm.

Fig 14 represents the better results of the Light gradient boosting machine with grid search hyperparameter optimization as a classification report in the form of precision, recall and support report with a graphical representation of ROC and confusion matrix.

Table 10 represents the optimized parameters of the Decision tree using various optimization algorithms. Additionally, Table 10 illustrates the improved results of the Decision tree with the genetic algorithm optimization compared to other hyperparameter optimization algorithms.

**Table 10:** Results of Decision tree.

S.no	Hyperparameter optimization Algo	Accuracy	Optimized parameter	Computation Time
1	Default Hyperparameters	0.9515469	{random_state = 5,max_depth = 12,criterion = ‘ginni’}	80.5s
2	Grid Search	0.94546913	{random_state = 8,max_depth = 19,criterion = ‘entropy’}	156.06s
3	Random Search	0.951765	{random_state = 25,max_depth = 22,criterion = ‘ginni’}	107.89s
**4**	**Genetic Algorithm**	**0.9566811**	**{random_state = 42,max_depth = 7,criterion = ‘entropy’}**	**135.8s**
5	Bayesian Optimization	0.94684513	{random_state = 5,max_depth = 12,criterion = ‘entropy’}	230.5s

**Fig 15:**
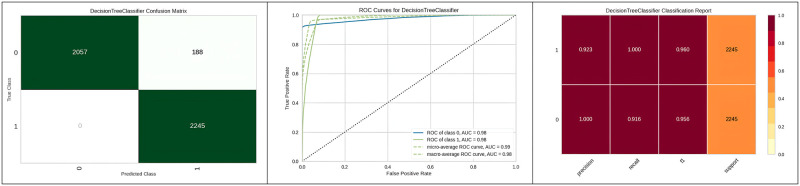
Best optimizer results of Decision tree.

Fig 15 represents the confusion matrix of the model, ROC curve and classification report of SVM with genetic algorithm hyperparameter optimization.

Table 11 represents the outcomes following the hyperparameter optimization process. The results demonstrate that the genetic algorithm for hyperparameter optimization in the KNN gives better results than other algorithms considered for the same purpose.

**Table 11:** Results of KNN.

S.no	Hyperparameter optimization Algo	Accuracy	Optimized parameter	Computation Time
1	Default Hyperparameters	0.830099	{’n_neighbors’. 5}	60.5s
2	Grid Search	0.82604116	{’n_neighbors’. 20}	176.06s
3	Random Search	0.830941	{’n_neighbors’. 11}	147.89s
**4**	**Genetic Algorithm**	**0.8354435**	**KNeighborsClassifier__n_neighbors = 17**	**167.8s**
5	Bayesian Optimization	0.8265757	{’n_neighbors’. 13.0}	350.5s

**Fig 16:**
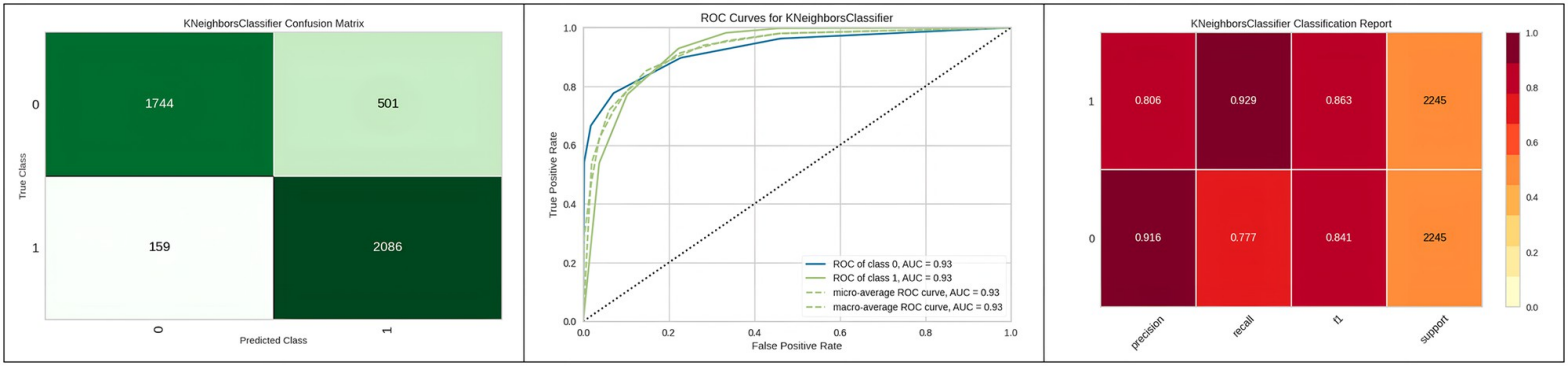
Best optimizer results of KNN.

Fig 16 represents the ROC curve and classification value report of KNN with the best hyperparameter.

Pondering further, the dataset was reduced using the Binary Grey wolf feature selection technique to find the most relevant features. The dataset with selected features was then used for further testing. Table 12 represents the results of the gradient boosting classifier using a feature selection dataset. The results in Table 12 clearly show the outperformance of the gradient boosting classifier with the Genetic algorithm of hyperparameter optimization.

**Table 12:** Results of gradient boosting classifier.

S.no	Hyperparameter optimization Algo	Accuracy	Optimized parameter	Computation Time
1	Default Hyperparameters	0.95072440	{’max_depth’. 3,’n_estimators’.100,’random_state’. none, ’learning_rate’. 0.1, ’subsample’. 1.0}	210.5s
**2**	**Grid Search**	**0.9564311**	**{’learning_rate’. 0.1, ’n_estimators’. 2000, ’random_state’. 1, ’subsample’. 0.5, ’max_depth’. 1}**	**187.06s**
3	Random Search	0.95191281	{’max_depth’. 4, ’subsample’. 0.5, ’random_state’. 1, ’n_estimators’. 1000, ’learning_rate’. 0.01}	57.89s
4	Genetic Algorithm	0.9492614	{’max_depth’. 1, ’subsample’. 0.75, ’random_state’. 1, ’n_estimators’. 1897, ’learning_rate’. 0.01}	298.8s
5	Bayesian Optimization	0.95081281	{’max_depth’. 1, ’subsample’. 0.75, ’random_state’. 1, ’n_estimators’. 1897, ’learning_rate’. 0.01}	380.5s

**Fig 17:**
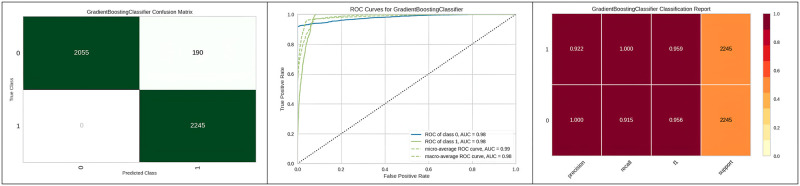
Best optimizer results of gradient boosting classifier.

Fig 17 presents the confusion matrix, ROC curve and classification report obtained after performing hyperparameter optimization using the algorithm on the Gradient boosting classifier.

**Fig 18:**
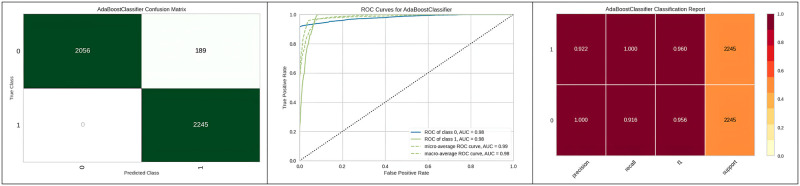
Best optimizer results of Adaboost.

Table 13 represents the results of Adaboost with a genetic algorithm, demonstrating high accuracy values. Additionally, Fig 18 represents the confusion matrix, classification report and ROC curve to represent the accuracy of the best optimizer’s results on Adaboost. The feature selection technique reduces the execution time of the Extra tree with random search hyperparameter optimization with the Genetic Algorithm.

**Table 13:** Results of Adaboost.

S.no	Hyperparameter optimization Algo	Accuracy	Optimized parameter	Computation Time
1	Default Hyperparameters	0.9505268	learning_rate = 1.0, n_estimators = 50, algorithm = ’SAMME.R’,	210.5s
2	Grid Search	0.951927292	{’learning_rate’. 1.0, ’algorithm’. ’SAMME.R’, ’n_estimators’. 20}	198.06s
3	Random Search	0.9498329	{’learning_rate’. 0.97, ’n_estimators’. 20, ’algorithm’. ’SAMME.R’}	126.89s
**4**	**Genetic Algorithm**	**0.9565291**	**{’learning_rate’. 1.02, ’algorithm’’n_estimators’. 10,. ’SAMME.R’}**	**194.8s**
5	Bayesian Optimization	0.94962440	{’learning_rate’. 0.82, ’n_estimators’. 8, ’algorithm’. ’SAMME’}	230.5s

**Fig 19:**
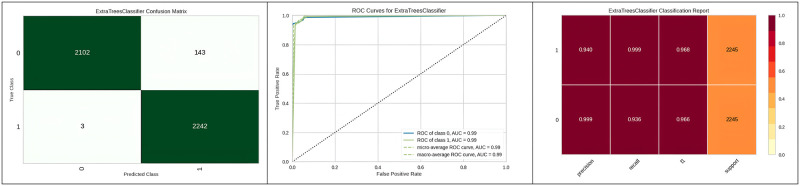
Best optimizer results of Extra tree.

Table 14 represents the results of hyperparameter optimization on the Extra tree. Fig 19 represents the ROC curve and classification report representing the results of the best optimizer.

**Table 14:** Results of Extra tree.

S.no	Hyperparameter optimization Algo	Accuracy	Optimized parameter	Computation Time
1	Default Hyperparameters	0.95143	{n_estimators = 100, max_depth = None, min_samples_split = 2, criterion = ’gini’,}	160.5s
2	Grid Search	0.9562421	(min_samples_leaf = 10, n_estimators = 200, max_features = 3)	178.06s
3	Random Search	0.9579176	{’min_samples_leaf’. 5, ’max_features’. 4, ’n_estimators’. 100}	197.89s
**4**	**Genetic Algorithm**	**0.9664197**	**min_samples_leaf = 20, n_estimators = 100, max_features = 4**	**135.8s**
5	Bayesian Optimization	0.952421	min_samples_leaf = 10, n_estimators = 300, max_features = 3	260.5s

The findings of different hyperparameter optimization on light gradient boosting machine are compared to the results in Table 15, it’s clearly show that Lightbgm model has improved slightly in performance with the grid search hyperparameter optimization algorithm.

**Table 15:** Results of Lightbgm.

S.no	Hyperparameter optimization Algo	Accuracy	Optimized parameter	Computation Time
1	Default Hyperparameters	0.94951234	(learning_rate = 0.1, num_leaves = 31, max_depth = 1, n_estimators = 100, reg_lambda = 0.0, colsample_bytree = 1.0)	160.5s
**2**	**Grid Search**	**0.9564825**	**(colsample_bytree = 0.6, max_depth = 5, num_leaves = 5, reg_lambda = 1, learning_rate = 1, n_estimators = 20)**	**178.06s**
3	Random Search	0.9501229	{’max_depth’. 10, ’reg_lambda’. 0.1, ’num_leaves’. 50, ’n_estimators’. 20, ’colsample_bytree’. 1, ’boosting_type’. ’dart’, ’learning_rate’.0.1}	197.89s
4	Genetic Algorithm	0.9490135	(learning_rate = 0.1,boosting_type = gbdt,colsample_bytree = 0.6, max_depth = 10, num_leaves = 50, reg_lambda = 0.1, n_estimators = 35)	135.8s
5	Bayesian Optimization	0.940296	(colsample_bytree = 0.6,, max_depth = 15, n_estimators = 35, num_leaves = 5, reg_lambda = 1, learning_rate = 1)	260.5s

**Fig 20:**
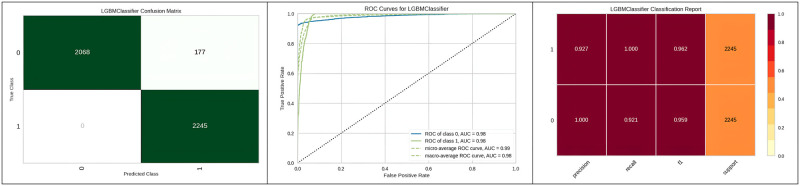
Best optimizer results of Lightbgm.

Fig 20 represents the confusion matrix and ROC curve of the Lightbgm classifier with Grid search hyperparameter optimization.

Table 16 represents the results of Random forest with different hyperparameter tuning algorithms with different parameters of random forest. Furthermore, the results of a Random Forest with Bayesian Optimization, demonstrate high accuracy values.

**Table 16:** Results of Random Forest.

S.no	Hyperparameter optimization Algo	Accuracy	Optimized parameter	Computation Time
1	Default Hyperparameters	0.957521518	max_depth = 2, random_state = 0	10.5s
2	Grid Search	0.95905372	{’criterion’. ’entropy’, ’max_depth’. 50, ’n_estimators’. 30}	6.06s
3	Random Search	0.9513258	{’min_samples_leaf’. 8, ’criterion’. ’gini’, ’max_features’. 35, ’min_samples_split’. 9, ’n_estimators’. 66, ’max_depth’. 42}	7.89s
4	Genetic Algorithm	0.9589267	min_samples_split = 3,’criterion’. ’gini’,max_depth = 17, min_sample s_leaf = 10, n_estimators = 120, max_features = 32	15.8s
**5**	**Bayesian Optimization**	**0.963746**	**{’min_samples_leaf’. 10.0, ’criterion’. 0, ’max_depth’. 43.0, ’min_samples_split’. 3.0, ’n_estimators’. 17.0’,max_features’. 10.0}**	**30.5s**

**Fig 21:**
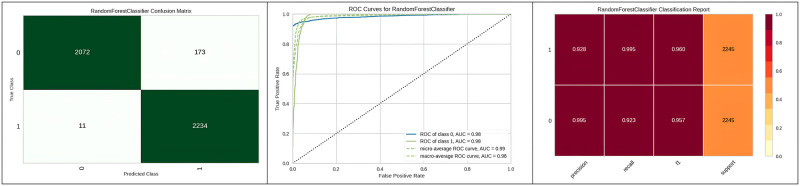
Best optimizer results of Random Forest.

Fig 21 represents the confusion matrix, ROC curve and classification report of Random forest with the Genetic Algorithm hyperparameter optimization.

However, the efficacy of both the Decision tree and KNN models has significantly improved with genetic algorithm by 0.13% in Tables 17 and 18.

**Table 17:** Results of Decision tree.

S.no	Hyperparameter optimization Algo	Accuracy	Optimized parameter	Computation Time
1	Default Hyperparameters	0.9591079	{random_state = 5,max_depth = 12,criterion = ‘ginni’}	80.5s
2	Grid Search	0.9445469	{random_state = 5,max_depth = 15,criterion = ‘entropy’}	116.06s
3	Random Search	0.9574765	{random_state = 5,max_depth = 58,criterion = ‘ginni’}	127.89s
**4**	**Genetic Algorithm**	**0.96884513**	**{random_state = 42,max_depth = 80,criterion = ‘ginni’}**	**135.8s**
5	Bayesian Optimization	0.96084513	{random_state = 5,max_depth = 32,criterion = ‘entropy’}	230.5s

**Table 18:** Results of KNN.

S.no	Hyperparameter optimization Algo	Accuracy	Optimized parameter	Computation Time
1	Default Hyperparameters	0.86430099	{’n_neighbors’. 5}	60.5s
2	Grid Search	0.85604116	{’n_neighbors’. 15}	176.06s
3	Random Search	0.873094	{’n_neighbors’. 09}	147.89s
**4**	**Genetic Algorithm**	**0.8877094**	**KNeighborsClassifier__n_neighbors = 17**	**167.8s**
5	Bayesian Optimization	0.8655757	{’n_neighbors’. 8.0}	350.5s

**Fig 22:**
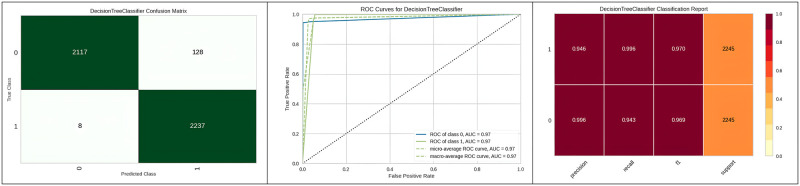
Best optimizer results of Decision tree.

Fig 22 represents the confusion matrix and ROC curve of the Decision tree classifier with Genetic Algorithm hyperparameter optimization.

Furthermore, Table 18 represents the better performance of the KNN algorithm with genetic algorithm hyperparameter optimization. The results clearly indicate that the algorithm performed with 17 neighbors gives better results.

**Fig 23:**
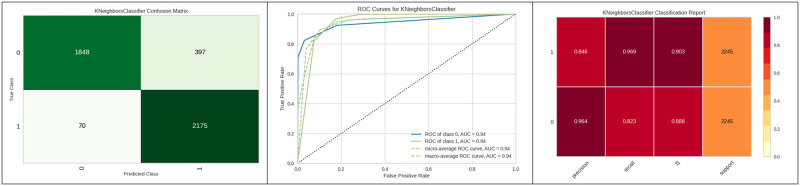
Best optimizer results of KNN.

Fig 23 represents the confusion matrix of the model, ROC curve and classification report of KNN with genetic algorithm hyperparameter optimization.

Moving forward, using the top hyperparameters and a feature-selected dataset, an ensemble model is created. Evaluation and comparison of the best combination of machine learning algorithms compared with other machine learning algorithms. The ensemble model is created by finding the best combination of models with hyperparameter optimization algorithm parameters on the feature selection dataset. The Random forest, Adaboost and KNN model combination perform best as compared to other models. The accuracy of the ensemble model is 98% which is better than the hyperparameter optimized machine learning algorithm.

**Fig 24:**
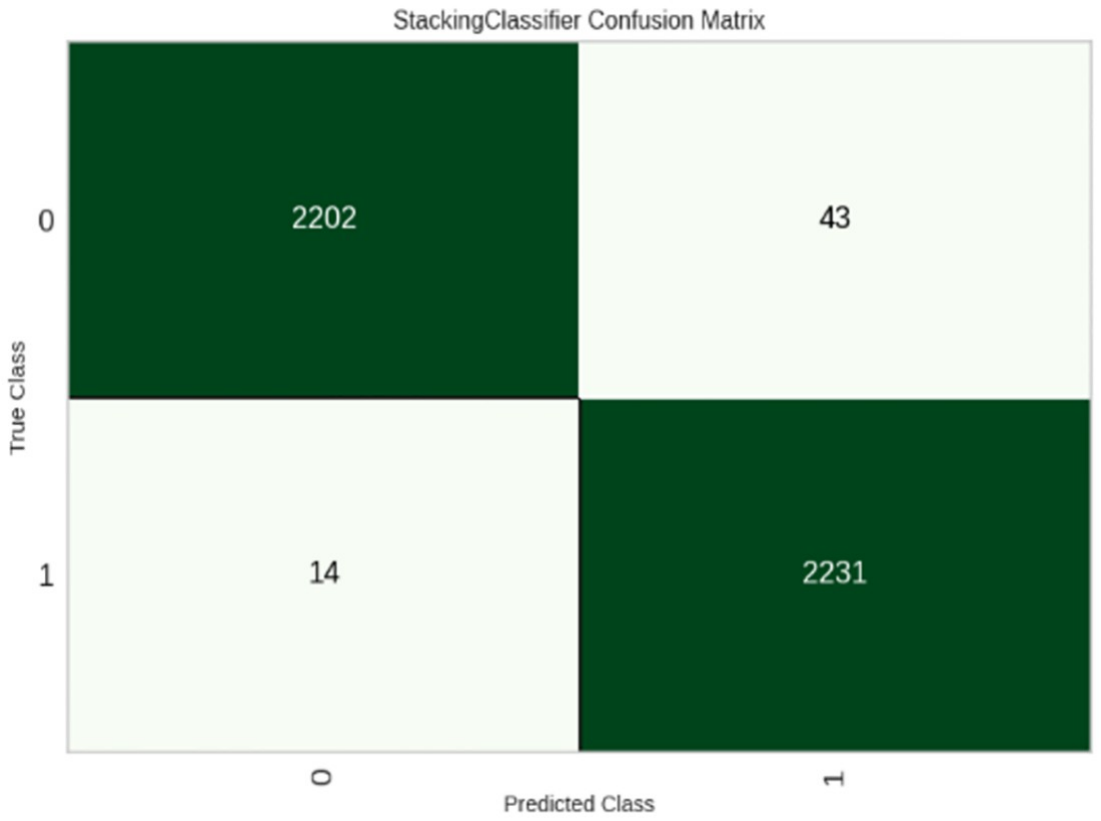
Confusion matrix of ensemble model.

Fig 24 implies that the ensemble model is capable of distinguishing between positive and negative COVID-19 diagnoses. The findings show that the methods used to create the ensemble model result in more precise and reliable classification.

The ensemble model findings show that combining HPO-KNN, HPO-Random Forest, and HPO-Adaboost improves model performance when compared to other models in the trial. The results show that the feature selection method improves the model success rate on COVID-19 dataset records. As a result, the model trains have more qualified data, which improves efficiency. Furthermore, the use of HPO-KNN, HPO-Random Forest, and HPO-Adaboost improves model stability. Furthermore, because it incorporates predictions from various classification models, the fundamental blocks of the ensemble classification model produce a robust prediction.

Table 19 represents the comparison of the Ensemble model with other machine learning algorithms. The results are compared with different evaluation metrics like accuracy, recall, precision and f-measure.

**Table 19:** Comparison table of the proposed model.

Model	Accuracy	Recall	Precision	F-measure
Decision Tree Classifier	0.9593	0.9926	0.9484	0.9700
Extra Trees Classifier	0.9564	0.9935	0.9425	0.9673
Random Forest Classifier	0.9544	0.9962	0.9367	0.9655
Gradient Boosting Classifier	0.9489	0.9948	0.9283	0.9604
Light Gradient Boosting Machine	0.9484	0.9977	0.9250	0.9600
Ada Boost Classifier	0.9475	0.9877	0.9200	0.9583
K Neighbors Classifier	0.8300	0.8862	0.7967	0.8390
**Ensemble model**	**0.9804**	**0.9994**	**0.9865**	**0.9898**

**Fig 25:**
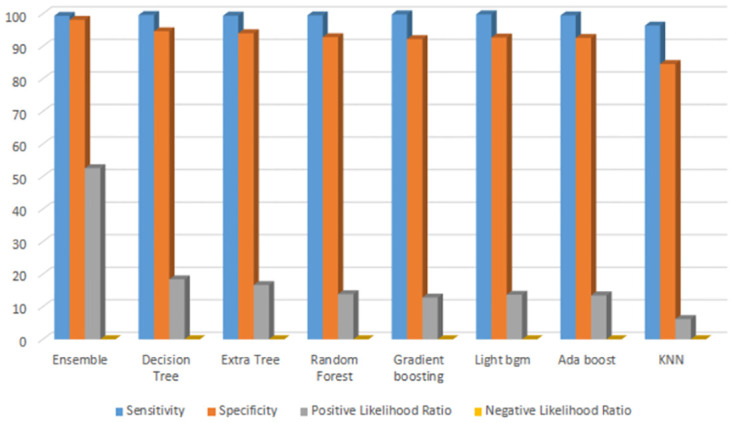
Statistical analysis of proposed model with other model.

Fig 25 shows the statistical analysis of the proposed model compared with other machine learning models. The graph displays the sensitivity, specificity, positive likelihood, and negative likelihood of each model.

**Fig 26:**
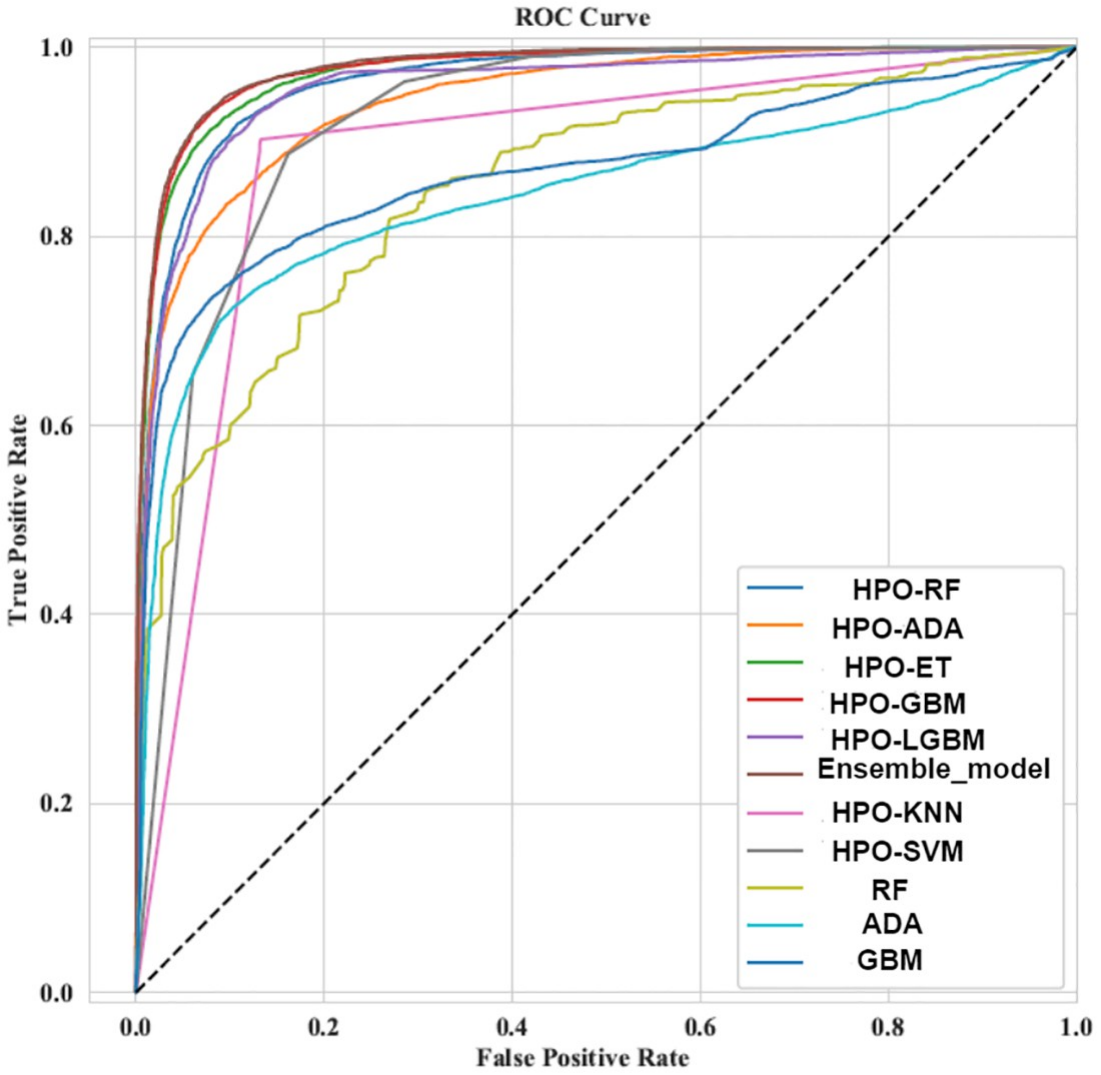
ROC comparison of machine learning algorithm.

Fig 26 represents a graphical depiction of the ROC demonstrating that the ensemble methods takes the peak in terms of ACC, while the GBM stays at the bottom. Table 20 represents the comparison of related studies in predicting COVID-19 and the proposed model which clearly indicate maximum accuracy with an ensemble of three machine learning algorithms.

**Table 20:** Comparison of related studies in predicting COVID-19 diagnosis.

Reference	Dataset Source	Model Used	Maximum Accuracy
Muhammad et al. [60]	263,007 patients from Mexico	Five models	95%
Han et al. [61]	375 patients from Wuhan	Broad Learning System	95%
Bruce Bode et al. [62]	398 patients from Texas	Many models	91%
Krishnaraj et. al. [63]	263,007 patients from Mexico	Five Ensemble Algorithm	96%
Aldonso et al. [64]	179,098 COVID-19	Many models	90%
**Proposed Model**	**14964 patients from Mexico**	**Three Algorithm Ensemble**	**98.04%**

## 7. Feature importance using Explainable AI (SHAP analysis)

AI solutions were black box in nature, necessitating model explanation. If machine learning experts develop tools to comprehend and explain the models they constructed, non-technical people’s doubts and suspicions are legitimate. SHAP [5] is one of the tools that was introduced a few years ago. It can deconstruct any machine learning model or deep neural net to make them intelligible to everyone. SHAP analysis explains what (and how) different factors impact your model’s decisions. The significance of incoming characteristics in forecasting a target variable is represented by feature importance [52]. Most significantly, the listing of feature significance improves the predictive modeling project’s efficacy and efficiency. In this research, we used the SHAP summary image [53].

Explainable AI (XAI) models and methods include Decision Trees, Logistic Regression, and Rule-Based Models for intrinsic interpretability, and post-hoc techniques like LIME, SHAP, and Grad-CAM for explaining complex models. Other approaches like Counterfactual Explanations and Partial Dependence Plots provide global and local insights, while advanced models such as Explainable Boosting Machines and Bayesian Rule [54] Lists combine transparency with predictive power. These XAI techniques enhance trust and accountability in AI by making their decisions understandable and transparent. As we can see, using the SHAP summary represented in Fig 26 has two advantages. feature ordering and the impact of each feature [55].

The feature ranking in decreasing sequence is determined by the location on the y-axis. (Higher importance to lower importance). X-axis SHAP values [56] decide the impact of each feature; positive SHAP values demonstrate a direct link with the target variable, and the opposite is also true. Additionally, the red shading corresponds to higher feature values, contrasting with the blue shading that stands for lower feature values. The irregular and intersecting lines suggest a sense of dispersion [57]. The importance of features for any categorization or forecast can be easily assessed by sorting the features in descending order, with the most important feature occupying the peak point. For example, as shown in Fig 23, visualizes in the form of a bar plot of the best 10 characteristics, with “INTUBADO “at the top. The following dominant characteristics are "INTUBADO", "EDAD", "ENTIDAD_RES", "ENTIDAD_NAC", "EMBARAZO", and so on. In comparison to the other characteristics depicted in the diagram, "DIABETES" stays hidden. Furthermore, as shown in Fig 23, increased "INTUBADO", "EDAD" and "ENTIDAD_RES" have a negative SHAP value, indicating a negative association.

**Fig 27:**
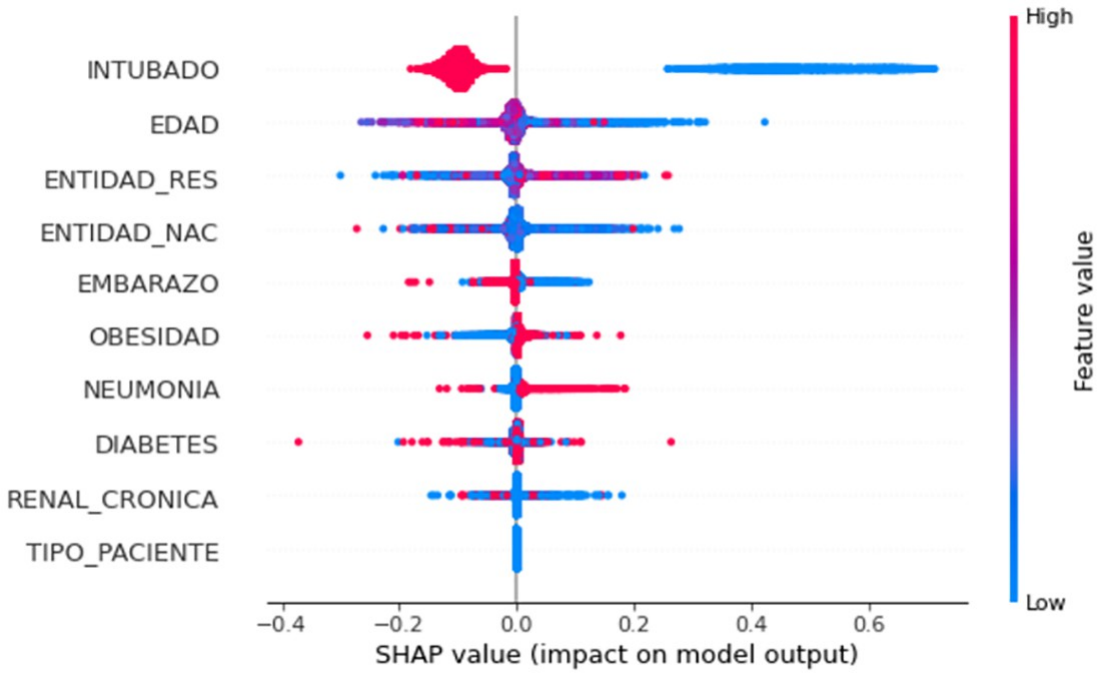
SHAP analysis.

Fig 27 represents the SHAP value of different features. It is obvious that greater values of this characteristic imply a lower probability of survival, i.e., in the case of COVID-19 here, and conversely as well. It’s important to highlight that the overview image provides a top-down view of the data [47]. The reliance plot for SHAP values and the feature interaction plot for SHAP values serve as tools to examine a particular feature and instance [48]. This assessment could determine how a sole feature influences the enhancement of model effectiveness, a matter not covered in this research, but reserved for future investigations.

**Fig 28:**
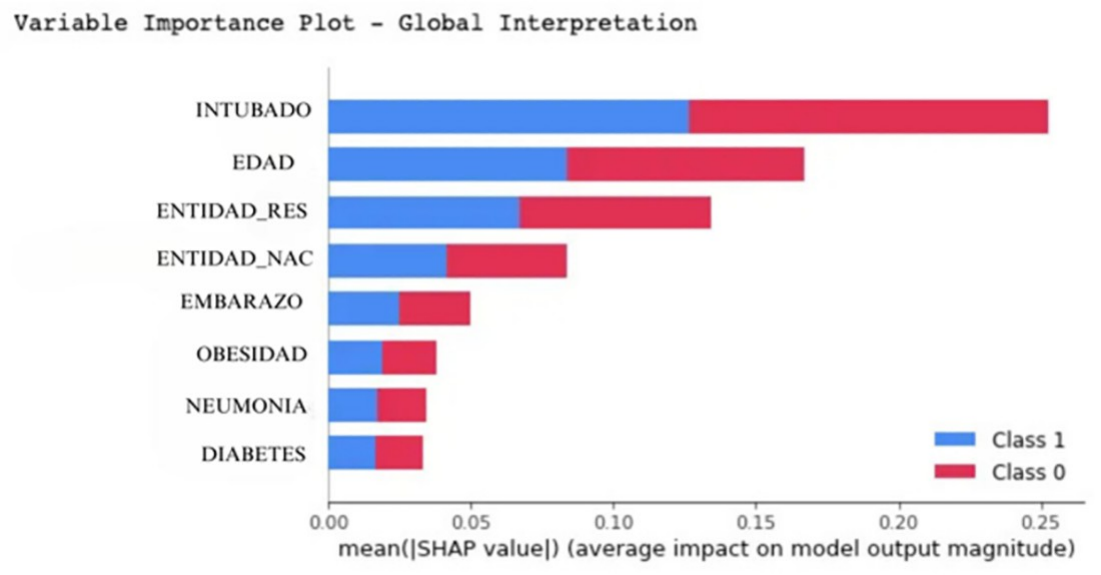
SHAP analysis mean value.

The Fig 28 illustrate the impact of features on the model’s predictions, distinguishing between Class 0 (COVID-19 positive) and Class 1 (COVID-19 negative). The graph highlights the significance of each variable with magnitude values, showcasing their importance in predicting COVID-19 outcomes [58]. This visualization effectively underscores the differential contribution of features in classifying COVID-19 status, facilitating a deeper understanding of the model’s decision-making process [23, 59].

## 8. Conclusions and future works

This study aspires to propose an integrated machine learning model for respiratory disease prediction taking into consideration one of the most fatal diseases recently seen by the human race that is COVID-19. Seven contemporary machine learning classifiers have been coupled with different hyperparameter optimization techniques and a feature selection approach with an aim to enhance the prediction capability. The system’s efficacy was rigorously evaluated through diverse performance metrics such as ACC, F1-score, MCC, and Kappa index, offering valuable insights from both patient and clinician viewpoints. The incorporation of SHAP values facilitates a comprehensive analysis of prediction outcomes for the observations. This technique of ranking input variables for identifying positive COVID-19 results helps to interpret the justification behind the model’s classification decisions. Furthermore, the proposed model can be readily extended to predict other ailments like diabetes, asthma, and hypertension. In summary, this study not only contributes to the realm of respiratory disease prediction but also lays the foundation for broader applications in disease forecasting. The seamless amalgamation of machine learning techniques, clinical datasets, and optimization strategies offers a holistic approach that has the potential to revolutionize healthcare analytics. Although this study is limited by the absence of experiments involving datasets with missing values and the evaluation of model performance on big data. Future work could explore the development of the model into an application integrated with Internet of Things (IoT) technologies. Furthermore, the work can be extended to utilize deep learning models for the extraction of features from the images and using standard machine learning techniques for classification while considering various evolutionary algorithms for feature selection.
